# A meta-analysis of periodic and aperiodic electrophysiological features in Parkinson’s disease

**DOI:** 10.1093/braincomms/fcag329

**Published:** 2026-08-28

**Authors:** Hamzeh Norouzi, Magdalena Ietswaart, Jason Adair, Gemma Learmonth

**Affiliations:** School of Psychology, University of Stirling, Stirling FK9 4LA, Scotland, UK; School of Psychology, University of Stirling, Stirling FK9 4LA, Scotland, UK; School of Computing Science and Mathematics, University of Stirling, Stirling FK9 4LA, Scotland, UK; School of Psychology, University of Stirling, Stirling FK9 4LA, Scotland, UK

**Keywords:** alpha oscillations, beta oscillations, EEG, MEG, specparam

## Abstract

Parkinson’s disease is characterized by a range of motor and non-motor changes that can negatively impact quality of life. Many studies have identified potential clinical electrophysiological correlates of Parkinson’s disease with an aim of developing new methods of identifying at-risk patients and forming the basis of therapeutic interventions. However, these studies do not present consistent results, and a formal meta-analysis is warranted to identify reliable EEG or magnetoencephalography (M/EEG) characteristics across datasets. In this meta-analysis of open-access M/EEG datasets (*n* = 6; 4 EEG and 2 MEG), we compared periodic and aperiodic characteristics of resting-state recordings in 368 patients with Parkinson’s disease and 570 age-matched healthy controls. Specifically, we compared the power and peak frequency of unadjusted and aperiodic-adjusted alpha- and beta-band oscillations, and the exponent and offset, across the two groups. Parkinson’s patients had a consistently elevated aperiodic exponent and offset. Patients also had higher unadjusted beta-band power, but this was no longer present when the aperiodic features were removed, suggesting that previous findings of elevated beta activity in Parkinson’s disease could be confounded by aperiodic activity rather than reflecting true oscillatory differences. Patients also had higher adjusted and unadjusted alpha-band power and a slower alpha peak frequency compared with controls, whereas no group difference in beta peak frequency was identified. In conclusion, this large cohort meta-analysis points to a broadly consistent pattern of both periodic and aperiodic changes in Parkinson’s patients in M/EEG signals that may be used to develop diagnostics and targeted interventions in the future.

## Teaser

Norouzi *et al*. investigated periodic and aperiodic resting-state features in Parkinson’s patients compared to controls, across six EEG and magnetoencephalography datasets. They found elevated alpha power, slower alpha peak frequency, and higher aperiodic offset and exponent in Parkinson’s patients. Aperiodic-adjusted analyses suggested beta differences were largely driven by aperiodic activity.

## Graphical Abstract

**Graphical Abstract:**
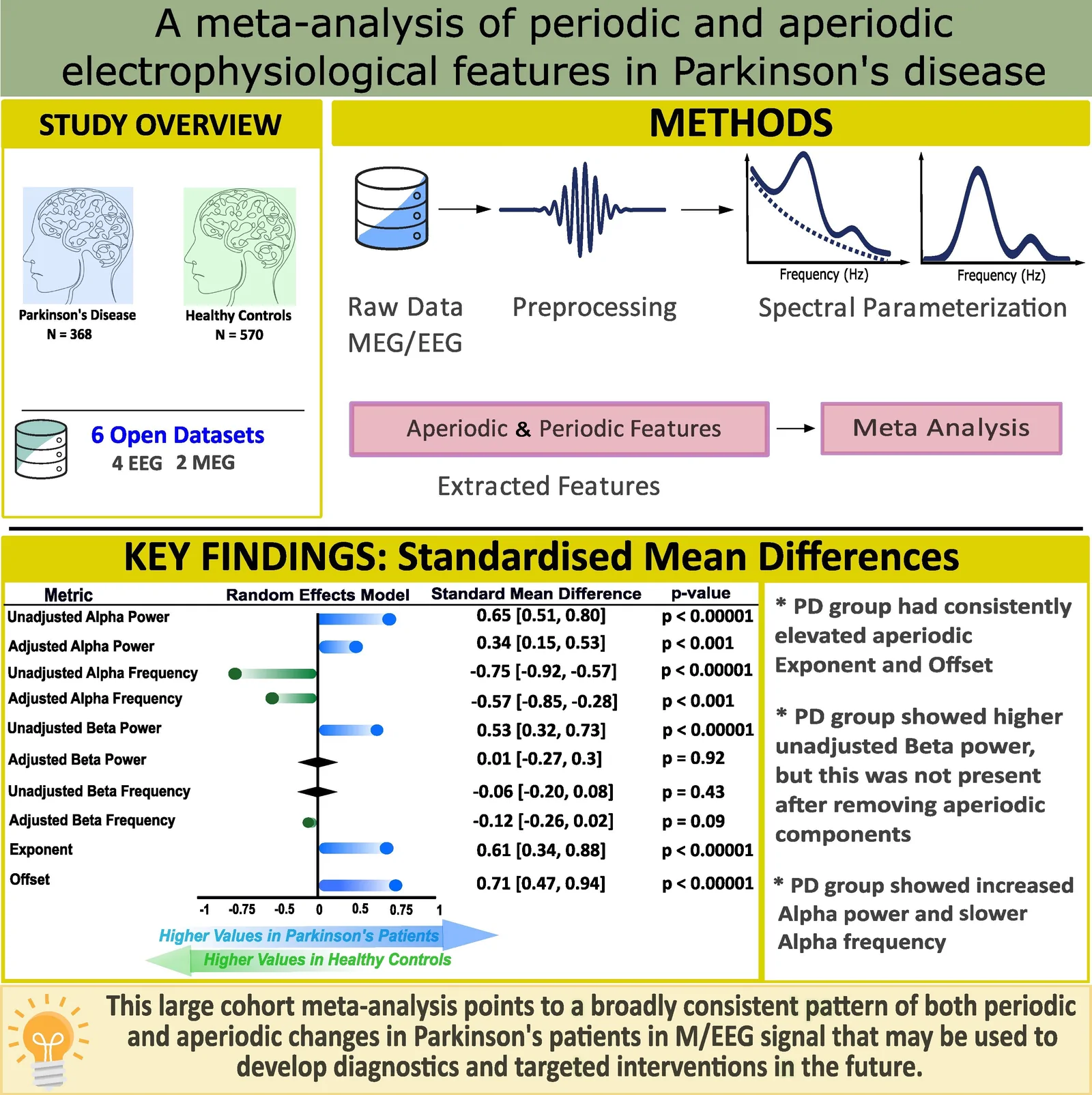

## Introduction

Parkinson’s disease is a complex and progressive neurodegenerative disorder, primarily affecting the execution of smooth movement, and is characterized by tremors, rigidity, bradykinesia and postural gait impairments.^1^ A typical neuropathological hallmark of Parkinson’s disease is abnormal aggregation of alpha-synuclein in the brain, which leads to dysfunction of the synapses, neuronal loss and the formation of Lewy bodies.^2^ Although dopaminergic neurons in the substantia nigra are particularly vulnerable, not all affected neurons in Parkinson’s disease are dopaminergic, and there is no absolute selectivity for any specific neurotransmitter group or anatomical region.^3,4^ As a result, the complex clinical manifestations of Parkinson’s disease may also arise from the involvement of non-nigral neuronal populations.^5^ Additionally, alpha-synuclein can propagate from affected to healthy neurons, contributing to disease progression,^2^ and manifesting a range of non-motor symptoms, including cognitive decline, fatigue, apathy, visuospatial disturbances and sleep disruption as well as motor symptoms.^1^ The progression of Parkinson’s disease symptoms correlates with the topographical spread of alpha-synuclein across anatomically interconnected brain areas, supporting the concept of trans-synaptic spreading of pathology.^6^

Electrophysiological recordings have identified specific changes in cortical oscillatory activity in patients with Parkinson’s disease, particularly within the theta (4–7 Hz), beta (13–30 Hz) and gamma (40+ Hz) bands.^7^ One of the most consistent markers of ‘oscillopathy’ in Parkinson’s disease is the beta activity associated with motor preparation and execution. During active movement in healthy individuals, beta power gradually desynchronizes in anticipation of an action, reaches its lowest point during movement execution, and then rapidly rebounds to a level above baseline after the movement ends.^8^ In contrast, this pattern is disrupted in Parkinson’s disease patients: beta desynchronization before and during movement is reduced, and the post-movement beta rebound is also reduced in magnitude.^9-11^

Elevated beta power has also been observed at rest in Parkinson’s disease, with higher levels when patients are off, compared with on, levodopa therapy. This elevation has been directly associated with motor dysfunction in the form of bradykinesia^12^ and freezing of gait.^13^ This has been corroborated by invasive electrophysiology, which has identified elevated resting-state beta oscillations within the basal ganglia nuclei and cortex.^14-16^ However, the literature is mixed, with non-invasive resting-state studies variously describing higher beta activity in Parkinson’s disease patients relative to controls,^17,18^ lower beta activity^19-21^ and no difference between patients and controls. Further, dopaminergic medication appears to effectively suppress beta activity at the cortical and subcortical levels.^14^ Thus, although beta oscillations may show some promise in dissociating Parkinson’s disease from controls, this potential is limited due to its sensitivity to physiological and pharmacological influences.

In contrast to beta-band abnormalities, lower frequency oscillations, particularly alpha activity (8–12 Hz), have been explored less often in Parkinson’s disease. In healthy adults, alpha rhythms typically reflect decreased excitability of the underlying cortex and are modulated by both tonic and selective attention.^22,23^ Several studies have reported an increase in alpha power and slowing of peak alpha frequency in Parkinson’s disease, with these changes positively associated with non-motor disease symptoms, particularly cognitive decline.^19,24-30^ However, this pattern is also inconsistent, with others reporting lower alpha, or no differences between patients and healthy controls (HCs).^21,31-33^

Electrophysiological signals reflect a combination of periodic (oscillatory) and aperiodic features, which are thought to arise from distinct neural mechanisms.^34-36^ The aperiodic feature carries physiological significance and exhibits dynamic modulation in response to factors such as age, cognitive state and task demands.^36^ The aperiodic feature can be characterized by two parameters: the exponent and the offset. The exponent is equivalent to the slope of the log-power spectrum and has been tentatively linked to the balance of underlying excitatory and inhibitory synaptic currents (E/I ratio),^37,38^ while the offset reflects a global shift in the broadband signal across all frequencies. When the aperiodic signal is not removed from the EEG or magnetoencephalography (M/EEG) signal, it can artificially inflate or reduce estimates of oscillatory power and lead to potential misinterpretation of results.^36^

Recent advances in spectral parameterization of M/EEG data have highlighted that previous studies of group differences in periodic oscillatory power may be confounded by changes in aperiodic electrophysiological features.^36,39^ By modelling and removing the offset and exponent prior to analysing periodic activity, it becomes possible to isolate more subtle changes in cortical activity that could point to biomarkers of Parkinson’s disease that have previously been obscured.

In this study, we leveraged large-scale open-source M/EEG resting-state datasets in patients with Parkinson’s disease and healthy controls. We applied a single, custom processing pipeline to perform a spectral parameterization reanalysis of the raw datasets, followed by 10 meta-analyses to synthesize the evidence for between-group differences in the unadjusted and aperiodic-adjusted power and peak frequency of alpha and beta oscillations, and the aperiodic exponent and offset between Parkinson’s disease patients and controls.

## Materials and methods

### Study selection

**Figure 1:**
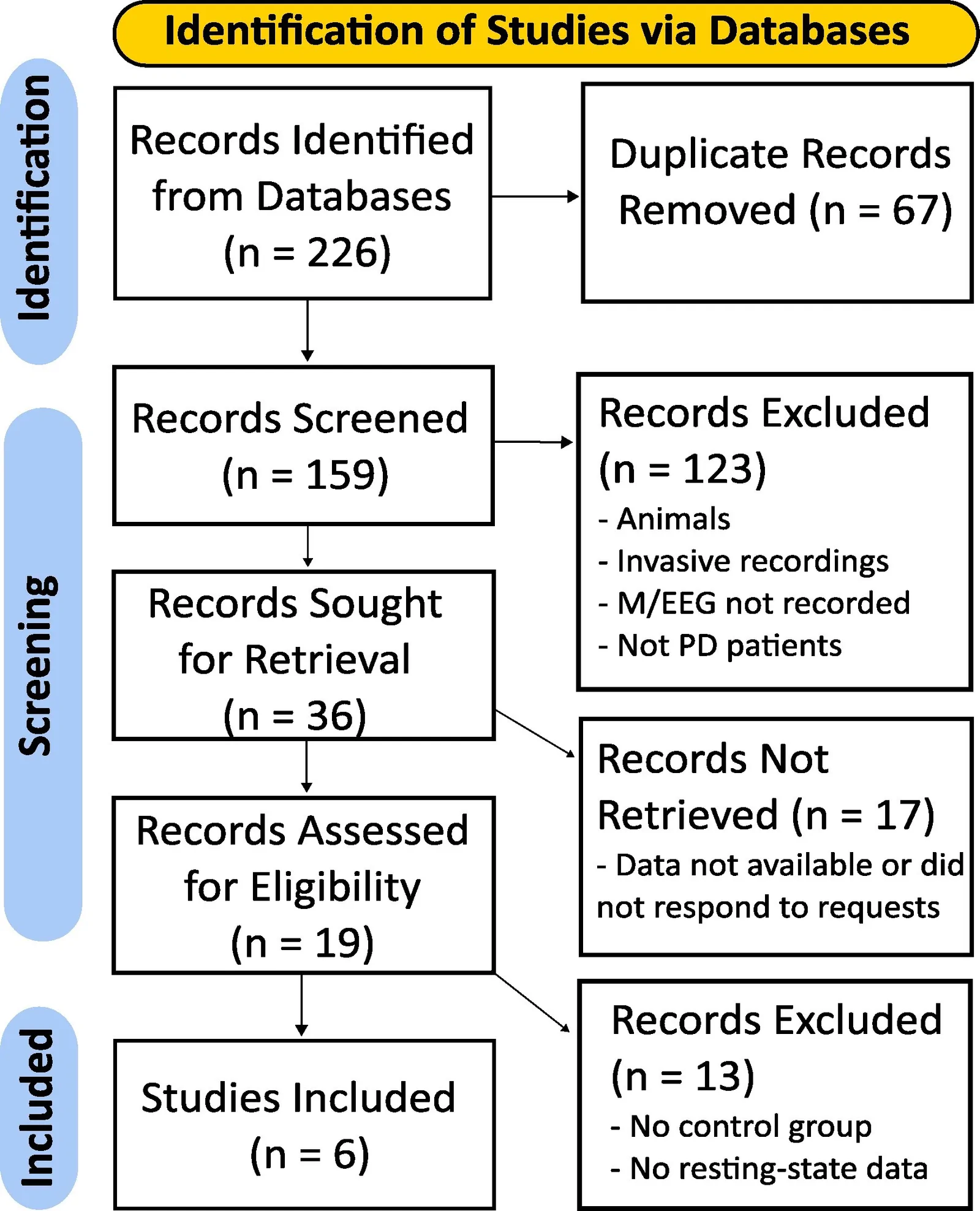
**Flow diagram documenting the electronic database searches, abstract screening and the reasons for excluding studies.** Adapted from Preferred Reporting Items for Systematic Reviews and Meta-Analyses (PRISMA^40^).

Studies were identified through electronic databases, including bioRxiv, medRxiv, PsyArXiv, PsycINFO, PubMed, ScienceDirect, Scopus and Web of Science from inception to September 2024. The search terms used to identify the datasets were (“Parkinsons’s disease” OR “Parkinsons” OR “Parkinsonian” OR “Parkinson”) AND (“resting state” OR “resting-state” OR “rest state” OR “rest-state” OR “rest”) AND (“EEG” OR “MEG” OR “electroencephalogram” OR “magnetoencephalogram”). We included datasets where raw resting-state EEG or MEG was available from both human Parkinson’s disease patients and healthy controls. A total of 226 studies were identified (Fig. 1). After removing duplicates, 159 unique records were screened and 123 records were excluded for the following reasons: did not include electrophysiological signal recordings, described animal models, recorded deeper brain structures using electrocorticography, local field potentials or deep brain stimulation (DBS), and investigated mild traumatic brain injury, multiple sclerosis or Parkinson’s disease patients during sleep. Of the 36 remaining records, 13 were further excluded because they only included patients, without a control group, or did not include resting-state data,^41-53^ and 17 records were not retrieved as they did not permit data sharing or did not respond to our data-sharing requests. Ultimately, resting-state data from six publicly available studies were included: San Diego,^54^ Turku,^55^ New Mexico^56^ and Iowa^57^ and two were shared with us upon request: OMEGA^58^ (the original report from the OMEGA dataset that we refer to is Wiesman *et al.*^29^ and NatMEG^59^). Details of the search are illustrated in Fig. 1.

The datasets entered into the preprocessing stage involved a total of 1092 participants, of which 427 participants were Parkinson’s disease patients, and 666 were healthy age-matched controls. The San Diego, Turku, Iowa and New Mexico datasets were recorded using EEG with different numbers and locations of sensors, and the OMEGA and NatMEG datasets were recorded using MEG. The San Diego, Turku and New Mexico datasets recruited patients both in ON and OFF medication states; however, we only included recordings for the ON medication state in this analysis. The characteristics of each dataset are presented in Table 1 and are described below.

**Table 1:** Characteristics of data cohorts assessing resting-state EEG/MEG in Parkinson’s patients and healthy controls

Study	Total participants	Participants included in meta-analysis	M/EEG	Resting state recording duration (min)	PD age	PD sex	Mean PD duration (years)	Mean H&Y	Mean UPDRS III score (ON medication)	HC age	HC sex
San Diego	16 PD, 16 HC	13 PD, 10 HC	EEG	>3	62.6 (8.3)	8F	4.9 (3.6)	–	33.7 (10.9)	63.5 (9.6)	9F
Turku	20 PD, 20 HC	6 PD, 20 HC	EEG	>2	69.5 (7.7)	10F	6 (5.0)	–	23.0 (10.2)	67 (6.1)	12F
Iowa	132 PD, 49 HC	115 PD, 42 HC	EEG	>2	68.1 (8.0)	43F	4.1 (3.5)	1.45	12.5 (7.2)	70.9 (7.6)	23F
New Mexico	27 PD, 25 HC	24 PD, 23 HC	EEG	≥2	69.7 (8.7)	9F	5.4 (4.1)	–	23.4 (9.9)	69.3 (9.6)	9F
OMEGA	166 PD, 488 HC	154 PD, 411 HC	MEG	≥5	65.0 (9.1)	42F	6.8 (4.3)	1.96 (0.7)	32.7 (14.8)	44.4 (20.8)	305F
NatMEG	66 PD, 68 HC	56 PD, 64 HC	MEG	≥3	64.4 (9.8)	22F	4.0 (3.4)	1.63 (0.77)	19.3 (11.1)	63.3 (8.7)	31F

Recording durations are reported as minimum values (‘≥’) because some participants had longer recordings, and in the OMEGA dataset multiple resting-state runs were concatenated.

PD = Parkinson’s disease, HC = Healthy controls, F = females, UPDRS = Unified Parkinson’s Disease Rating Scale, H&Y = Hoehn and Yahr Score.

### Dataset 1: San Diego, USA

Sixteen Parkinson’s disease patients (8 female, mean age = 62.6 years, SD = 8.3) and 16 HCs (9 female, mean age = 63.5 years, SD = 9.6) were recorded in the San Diego dataset.^54^ All patients had mild to moderate Parkinson’s disease severity (Hoehn and Yahr stage II and III). Parkinson’s disease patients were recorded both ON and OFF medication. EEG was recorded for at least 3 min using a 32-channel ActiveTwo (Biosemi Instrumentation system) at a sampling rate of 512 Hz while participants kept their eyes open and maintained fixation.

### Dataset 2: Turku, Finland

Twenty Parkinson’s disease patients (10 female, mean age = 69.5, SD = 7.7) and 20 controls (12 female, mean age = 67, SD = 6.1) were recorded in the Turku dataset.^55^ EEG was recorded for at least 2 min using 64 active sensors with a sampling rate of 500 Hz during eyes open and closed. Twelve patients were recorded OFF medication, and five patients were recorded ON medication, but only the eyes-open recordings were selected for the meta-analysis.

### Dataset 3: Iowa, USA

A total of 132 Parkinson’s disease patients (43 female, mean age = 68.1, SD = 7.98, all ON medication) and 49 controls (23 female, mean age = 70.91, SD = 7.62) were tested in the Iowa dataset.^57^ Forty-six-channel EEG was recorded with eyes open for at least 2 min with a sampling rate of 500 Hz.

### Dataset 4: New Mexico, USA

Twenty-five Parkinson’s disease patients (9 female, mean age = 69.68, SD = 8.73) and 25 age- and sex-matched controls (9 female, mean age = 69.32, SD = 9.58) were tested in the New Mexico dataset.^56^ EEG was recorded for around 9 min with 64 Ag/AgCl sensors at a sampling rate of 500 Hz, with both eyes closed and eyes open. Parkinson’s disease patients were tested both ON and OFF medication. The eyes-open, ON-medication data were used in the meta-analysis.

### Dataset 5: OMEGA, Canada

A total of 166 Parkinson’s disease patients (46 female, mean age = 64.95, SD = 9.12) and 488 HCs (307 female, mean age = 44.42, SD = 20.83) were tested in the OMEGA dataset.^58^ At least 5 min of eyes-open resting-state data was recorded using a 275-channel whole-head CTF MEG system at a sampling rate of 2400 Hz and with an antialiasing filter with a 600 Hz cut-off. The database was accessed in September 2024.

### **Dataset 6:** NatMEG**, Sweden**

Sixty-six Parkinson’s disease patients (28 female, mean age = 65.6, SD = 9.5) and 68 HCs (27 female, mean age = 63.93, SD = 8.4) were tested in the NatMEG dataset.^59^ At least 3 min of resting-state data was recorded using a Neuromag TRIUX 306-channel MEG system, with 102 magnetometers, with a 1000 Hz sampling rate, with an online 0.1 Hz high-pass ﬁlter and 330 Hz low-pass ﬁlter while the participants sat with their eyes closed. The average head movements were not signiﬁcantly different between groups (*t*(115.8) = 0.55, *P* = 0.58).

### Preprocessing

Data preprocessing and processing were conducted in MNE-Python.^60^ The analysis scripts are located at https://github.com/HamzehNorouzi/Meta_Reanalysis. The OMEGA, NatMEG and New Mexico datasets had previously been subjected to spectral parameterization, but for the purposes of this study, we developed a single processing pipeline in order to reanalyse the six datasets using a single set of common parameters. Although EEG and MEG measure different physical quantities and differ in absolute signal amplitude, both modalities produce power spectra with comparable frequency patterns. Because our analyses rely on parameterizing the shape of the power spectrum rather than comparing absolute power values between EEG and MEG, the scaling differences should not affect the estimation of the reported parameters, as standardized mean differences were calculated for the meta-analysis models. The processing steps were as follows:

### EEG datasets

Each of the EEG datasets was first resampled to a uniform sampling rate of 500 Hz, then filtered between 1 and 100 Hz using a zero-phase infinite impulse response (IIR) filter, then linearly detrended to remove slow drifts, and rereferenced to the average activity of all electrodes. Finally, independent component analysis (ICA), with the Infomax method, was applied to the signal and non-brain components were excluded automatically using MNE-ICALabel.^61^

### MEG datasets

Both gradiometer and magnetometer channels were available for the NatMEG dataset, but only magnetometers were available for the OMEGA dataset; therefore, only magnetometer channels were used for analysis of both MEG datasets. Similar to the EEG preprocessing, the MEG datasets were first resampled to 500 Hz and filtered between 1 and 100 Hz using a zero-phase IIR filter. To attenuate environmental magnetic noise, Signal Space Projection^62^ vectors were computed from empty-room data using the mne.compute_proj_raw() function in MNE-Python. This procedure identifies the dominant spatial patterns associated with environmental magnetic interference.^63,64^ These spatial filters were subsequently applied to each participant’s continuous MEG data using mne.apply_proj(). Next, ICA with the Infomax method was applied to the signal, and non-brain components were excluded automatically using MNE-ICALabel. For the OMEGA dataset, an additional step was included because the reference channels were available. Thus, to mitigate intermittent environmental noise in the OMEGA dataset, ICA was performed separately on the MEG sensors and the reference channels, enabling the identification and removal of both non-brain components as well as external noise sources.^65^

To further reduce artefacts, the preprocessed EEG and MEG signals were segmented into 1-s epochs, which resulted in each dataset having a different number of epochs due to the variable length of the recordings (mean and standard deviation of the number of epochs per dataset: San Diego: Parkinson’s disease = 189.0 ± 25.2, HC = 184.0 ± 11.0; Turku: Parkinson’s disease = 124.0 ± 24.8, HC = 91.5 ± 35.5; Iowa: Parkinson’s disease = 132.0 ± 42.3, HC = 148.0 ± 38.9; New Mexico: PD = 157.0 ± 12.2, HC = 165.0 ± 16.3; OMEGA: Parkinson’s disease = 1200.0 ± 216.5, HC = 481.0 ± 256.1; NatMEG: Parkinson’s disease = 190.0 ± 24.4, HC = 186.0 ± 19.7). Next, amplitude thresholds were automatically estimated using Autoreject^66^ based on the distribution of peak-to-peak values across epochs. Epochs exceeding these thresholds were rejected. Due to poor signal quality or data corruption, 9 subjects from the San Diego dataset, 23 subjects from Iowa, 5 subjects from New Mexico, 45 subjects from OMEGA and 11 subjects from NatMEG were excluded.

### Spectral parametrization

The power spectrum of each epoch was estimated using Welch’s method with a segment length of 5000 samples (10 × sampling rate), 50% overlap and a Hanning window using the mne.compute_psd() function, with no normalization applied either before or after spectral parameterization. Power spectra were then grouped into three regions of interest involving the frontal, central and posterior sensors, as well as an average across all sensors, referred to as the full scalp region. To group MEG sensors into frontal, central, and posterior regions, we used the 3D sensor coordinates and extracted each sensor's spatial position and assigned them to frontal, central and posterior groups (see Supplementary Fig. 11 for the MEG sensor layouts for each region of interest (ROI)). For the EEG datasets, frontal, central and posterior regions were assigned according to each sensor’s label (frontal and frontoparietal into the frontal region; central sensors into the central region; parietal, occipital and parieto-occipital into the posterior region).

Next, the power spectrum of each region was parameterized using the *specparam* toolbox^36^ in MNE-Python using the following settings: peak width limits ([0, 12]), peak threshold (1), aperiodic mode (fixed), maximum number of peaks (7) and minimum peak height (0.1). Each of the previously parameterized datasets had examined different frequency windows (from a lower bound of 0.5, 2 or 3 Hz to an upper bound of 40 Hz) and we therefore first parameterized the spectra across several broad and narrow frequency ranges to identify the optimal frequency windows and spatial locations for comparison across studies (see Supplementary Fig. 1). Through this process, we selected 3–40 Hz for the common pipeline.

**Figure 2:**
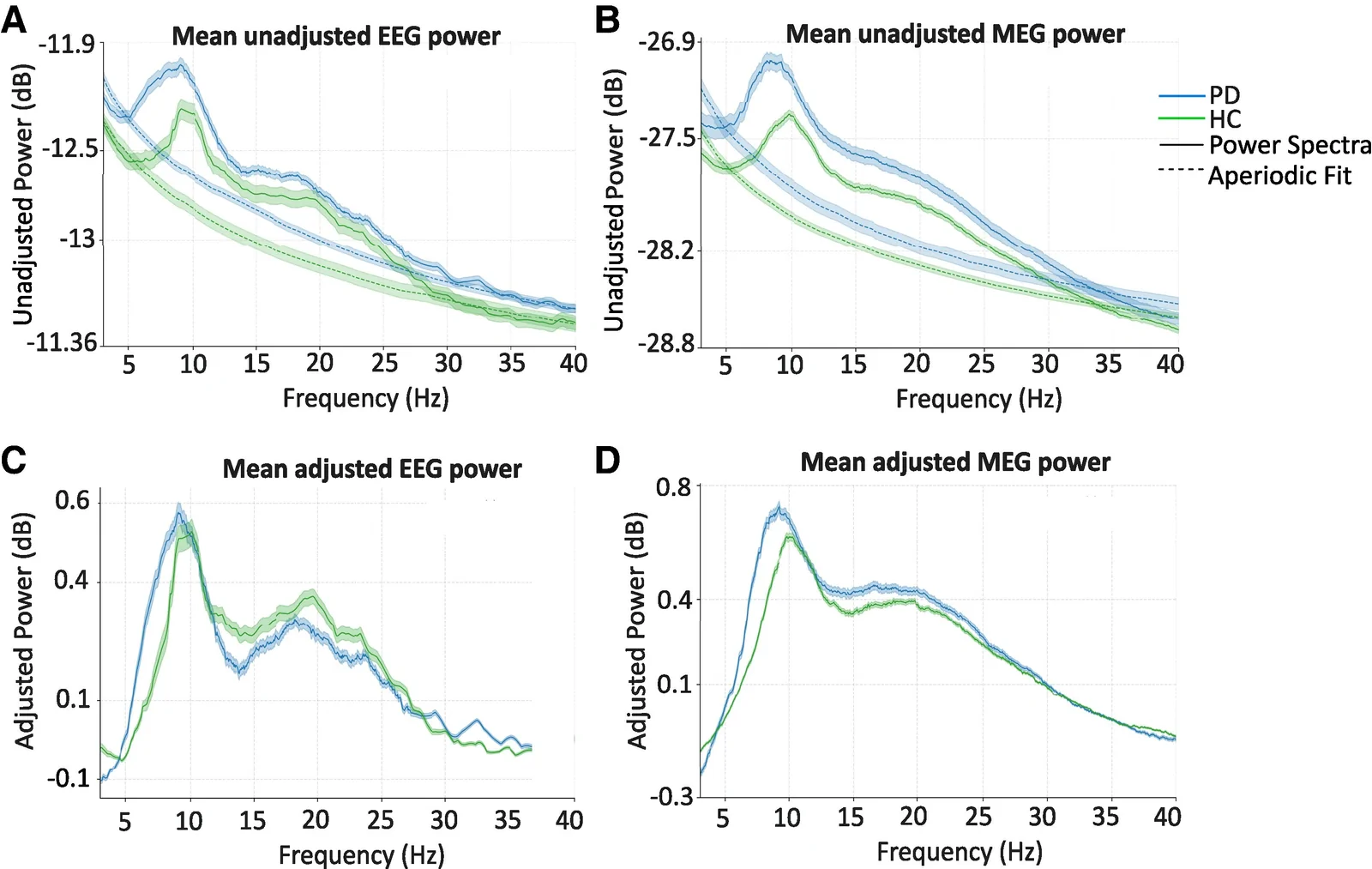
**Mean adjusted and unadjusted power.** (**A**) Mean unadjusted power (solid line) and aperiodic fit (dashed line) in the four EEG datasets. (**B**) Mean unadjusted power (solid line) and aperiodic fit (dashed line) in the two magnetoencephalography (MEG) datasets. (**C**) Mean adjusted power (aperiodic signal subtracted) in the four EEG datasets. (**D**) Mean adjusted power (aperiodic signal subtracted) in the two MEG datasets. For all plots, the power was spatially averaged across all sensors (in EEG or MEG) to obtain the full scalp mean power value, and shaded areas represent the standard error (see spectral parametrization section in the methods for details). The EEG datasets included 95 healthy controls and 158 Parkinson’s patients, while the MEG datasets comprised 475 healthy controls and 210 Parkinson’s patients.

The unadjusted and aperiodic-adjusted power spectra were used to extract the features of interest for meta-analysis. The unadjusted peak was estimated from the original spectrum as the highest peak value and its corresponding frequency within the alpha and beta ranges. The adjusted power was estimated by first subtracting the aperiodic signal, which was defined as:


$$L=b-\log\;(k+F^{x})$$

where *b* and *x* denote the offset and exponent, *F* is the frequency vector, and *k* is the knee parameter (set to zero).^36^ After aperiodic subtraction, the highest peak values and their respective peak frequencies within the alpha and beta ranges were extracted directly from this flattened spectrum. We did not use the built-in Gaussian peak-fitting outputs (i.e. peak power and central frequency) in *specparam*. To ensure a standardized comparison, both the unadjusted and adjusted peaks were estimated using a custom script designed to locate the peak power value and its corresponding frequency within the alpha and beta windows. These two frequency bands were selected because we observed clear peaks within the alpha (7–12 Hz) and beta (12–30 Hz) frequency windows through visual inspection of Fig. 2, but no peaks were identified within the theta or gamma bands. Other values, i.e. aperiodic exponent and offset, were obtained directly from *specparam*.

Error metrics were used to identify and exclude samples with poor model performance. For each data cohort, the model fit was obtained and the z-score of the model goodness-of-fit distribution was then computed to detect and exclude subjects with poor model performance. Model fits with *z*-values >2 were rejected. As a result, the following participants were excluded at this stage: 1 Parkinson’s disease patient from the San Diego dataset, 2 HCs from the Turku dataset, 4 Parkinson’s disease and 2 HCs from Iowa, 1 Parkinson’s disease and 2 HCs from New Mexico, 5 Parkinson’s disease and 14 HCs from OMEGA, and 3 Parkinson’s disease patients from NatMEG. A total of 938 subjects (368 Parkinson’s disease patients and 570 controls) were included in the meta-analysis stage. After removing outliers, the model accuracy had a mean fit of 97.04% (range = 96.54–97.8) for the Parkinson’s disease group and 97.38% (range = 96.98–97.7) for the HCs.

The data were collapsed over the full scalp, and the peak of unadjusted and adjusted alpha and beta power, and their respective adjusted and unadjusted peak frequencies, were entered into the meta-analysis, together with both aperiodic features, i.e. offset and exponent.

### Statistical analysis

The following features were extracted from each dataset and were used in the 10 meta-analyses: unadjusted peak alpha power, aperiodic-adjusted peak alpha power, unadjusted peak alpha frequency, aperiodic-adjusted peak alpha frequency, unadjusted peak beta power, aperiodic-adjusted peak beta power, unadjusted peak beta frequency, aperiodic-adjusted peak beta frequency, exponent and offset. The *metafor*^67^ and *robumeta*^68^ packages for R were used to perform the meta-analysis. First, the standardized mean difference and sampling variance were calculated using the *escalc* function between Parkinson’s patients and HCs, separately for each of the 10 features. For each of the analyses, an overall effect size estimate was calculated by weighting each feature effect size according to sample size, using a random-effects (RE) model. Mean weighted effect sizes and 95% confidence intervals (95% CIs) were estimated using restricted maximum likelihood (REML). Heterogeneity was assessed using the *Q*-statistic, *I*^2^ and tau^2^ indices. The *Q*-statistic tests whether the variability in effect sizes is greater than expected by sampling error alone. *I*^2^ identifies the proportion of variability attributable to true between-study differences. Tau^2^ estimates the absolute amount of between-study variance. Forest plots were used to visualize the standardized mean difference across datasets.

To investigate potential associations between patients’ age and the 10 periodic and aperiodic M/EEG features, while accounting for baseline differences across recording modalities, we used linear mixed-effects models (LMM).^69^ This approach enables estimation of a global age effect while controlling for study-specific offsets in signal magnitude arising from differences in the recording modality (MEG or EEG). Each model included age as a fixed effect and study as a random effect (random intercept). From a total of 368 samples from the Parkinson’s disease group, the patient’s age was missing from 27 samples. Therefore, 341 Parkinson’s disease samples from 6 different studies (95 samples from Iowa, 24 samples from New Mexico, 6 samples from Turku, 13 samples from San Diego, 49 samples from NatMEG and 154 samples from OMEGA) were used in the LMM analysis. Models were fitted using REML estimation. All LMM analyses were conducted in Python using the *Statsmodels* library.^70^

## Results

A total of 6 datasets involving 368 patients with Parkinson’s disease and 570 age-matched controls were included in meta-analyses of the (i) aperiodic exponent; (ii) aperiodic offset; (iii) unadjusted peak alpha power; (iv) aperiodic-adjusted peak alpha power; (v) unadjusted peak alpha frequency; (vi) aperiodic-adjusted alpha peak frequency; (vii) unadjusted peak beta power; (viii) aperiodic-adjusted peak beta power; (ix) unadjusted peak beta frequency and (x) aperiodic-adjusted peak beta frequency. The mean unadjusted and aperiodic-adjusted (i.e. aperiodic features subtracted) power are shown in Fig. 2, separately for Parkinson’s patients and HCs, and for MEG and EEG datasets (see also Supplementary Tables 1 and 2).

### Full scalp analysis

Please see Supplementary Tables 3–12 for full output of the RE models.

### Exponent

**Figure 3:**
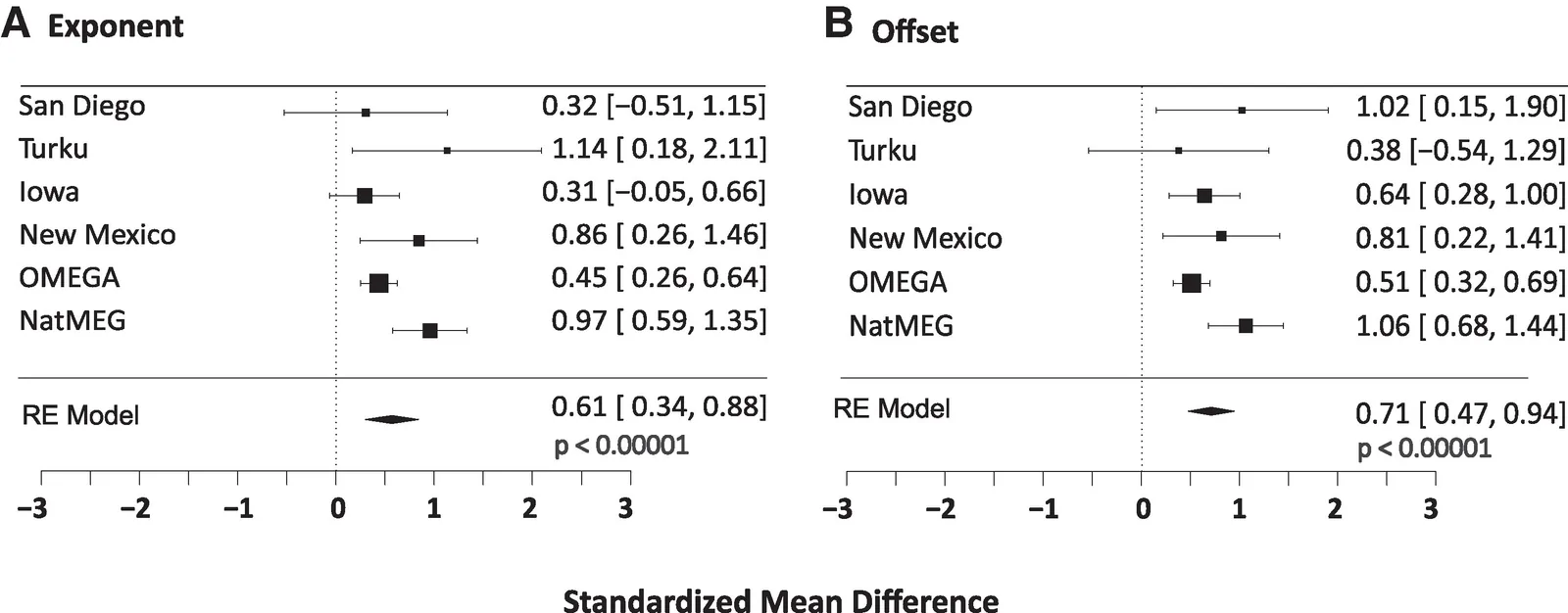
**Standardized mean differences in aperiodic features between Parkinson’s patients (*N* = 368) and healthy controls (*N* = 570). (A)** Aperiodic exponent. (**B)** Aperiodic offset. A positive number indicates values are higher in patients relative to controls, and a negative number indicates higher in controls relative to patients. Effect sizes for each feature were estimated using RE models with 95% CIs estimated using restricted maximum likelihood (REML).

Parkinson’s disease patients had a larger exponent compared with HCs: Standardized mean difference (SMD) = 0.61, 95% CI = [0.34, 0.88], *P* < 0.00001 (Fig. 3A), although there was evidence of heterogeneity: *Q*(5) = 10.40, *P* = 0.06, tau^2^ = 0.05, *I*^2^ = 55.47%, suggesting that effect sizes differed across the six datasets.

### Offset

Parkinson’s disease patients had a larger offset compared with controls: SMD = 0.71, 95% CI = [0.47, 0.94], *P* < 0.00001 (Fig. 3B) and there was no evidence of heterogeneity across datasets: *Q*(5) = 7.91, *P* = 0.16, tau*^2^* = 0.03, *I*^2^ = 41.87%, suggesting that effect sizes were consistent across the six included datasets.

### Unadjusted peak alpha power

**Figure 4:**
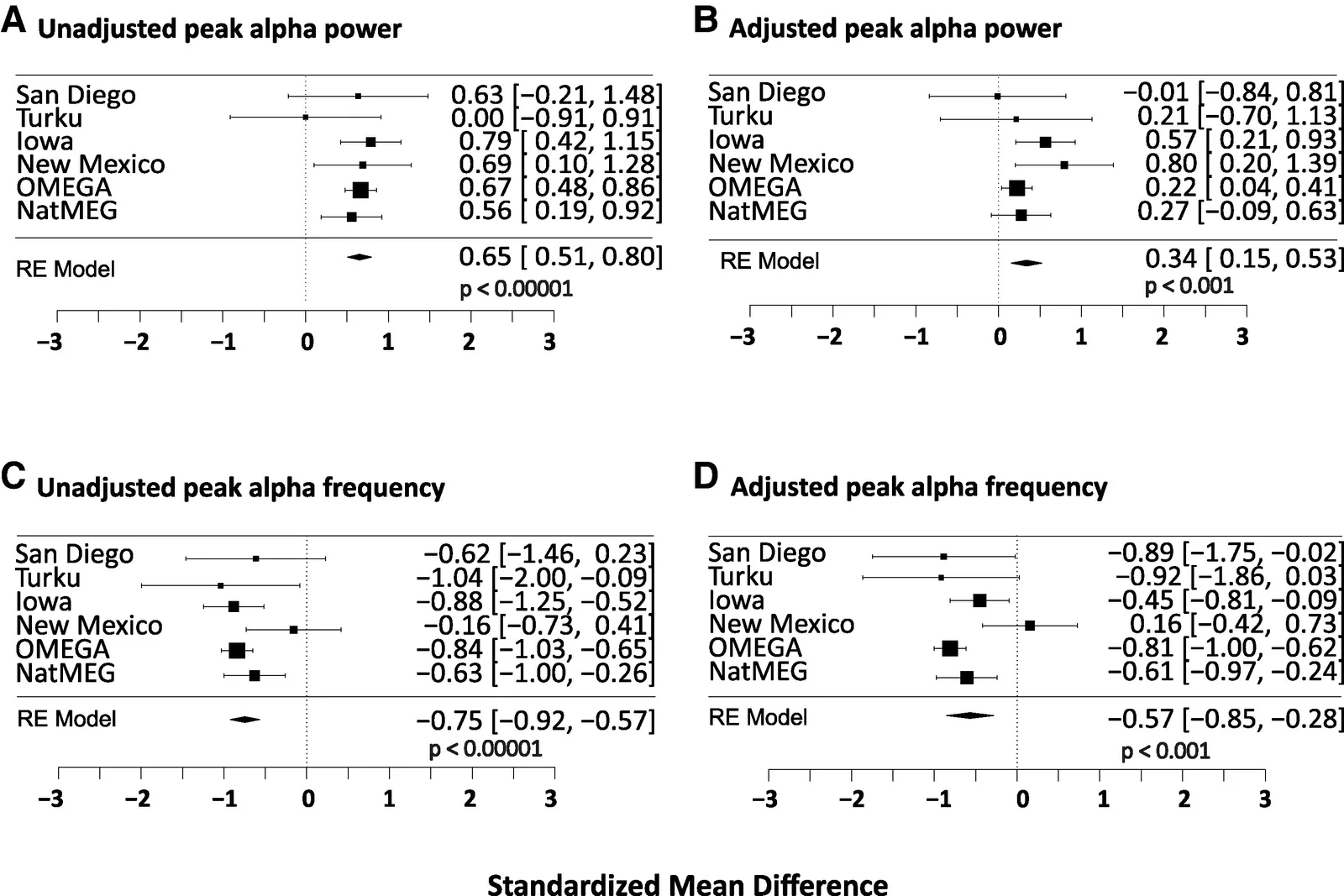
**Standardized mean differences in peak alpha power and peak alpha frequency between Parkinson’s patients (*N* = 368) and healthy controls (*N* = 570).** (**A**) Unadjusted peak alpha power. (**B**) Adjusted peak alpha power. (**C**) Unadjusted peak alpha frequency. (**D**) Adjusted peak alpha frequency. A positive number indicates values are higher in patients relative to controls, and a negative number indicates higher in controls relative to patients. Effect sizes for each feature were estimated using random-effects (RE) models with 95% CIs estimated using restricted maximum likelihood (REML).

An effect of group on unadjusted peak alpha power was observed, SMD = 0.65, 95% CI = [0.51, 0.8], *P*  *<* 0.00001, with a larger unadjusted alpha power for Parkinson’s disease patients relative to controls (Fig. 4A). There was no evidence of heterogeneity across the six datasets for unadjusted alpha power: *Q*(5) = 2.8, *P* = 0.72, tau^2^ = 0, *I*^2^ = 0%.

### Adjusted peak alpha power

When the aperiodic signal was subtracted from the original spectrum, the magnitude of the effect on alpha power, i.e. the adjusted alpha power, slightly reduced, although alpha power was still larger in Parkinson’s disease patients relative to controls, SMD = 0.34, 95% CI = [0.15, 0.53], *P* < 0.001 (Fig. 4B). There was no evidence of heterogeneity across the six datasets for adjusted alpha power: *Q*(5) = 6.05, *P* = 0.30, tau^2^ = 0.01, *I*^2^ = 22.50%, indicating that the effect size estimates were relatively stable across the six included studies.

### Unadjusted peak alpha frequency

There was an effect of group on unadjusted peak alpha frequency, SMD = −0.75, 95% CI = [−0.92, −0.57], *P* < 0.00001, with slower unadjusted alpha central frequency for Parkinson’s disease patients relative to controls (Fig. 4C). There was no evidence of heterogeneity across datasets: Q(5) = 6.24, *P* = 0.28, tau^2^ = 0.008, *I*^2^ = 15.76%, again indicating consistency of effect sizes across the six included studies.

### Adjusted peak alpha frequency

After subtracting the aperiodic signal from the original spectrum, the magnitude of the group difference in adjusted alpha peak frequency decreased; however, Parkinson’s disease patients still showed slower adjusted alpha peak frequency relative to controls, SMD = −0.57, 95% CI = [−0.85, −0.28], *P* < 0.001 (Fig. 4D). There was evidence of heterogeneity, *Q*(5) = 12.12, *P* = 0.033, tau^2^ = 0.066, *I*^2^ = 60.3%, suggesting that effect sizes differed across the datasets.

### Unadjusted peak beta power

**Figure 5:**
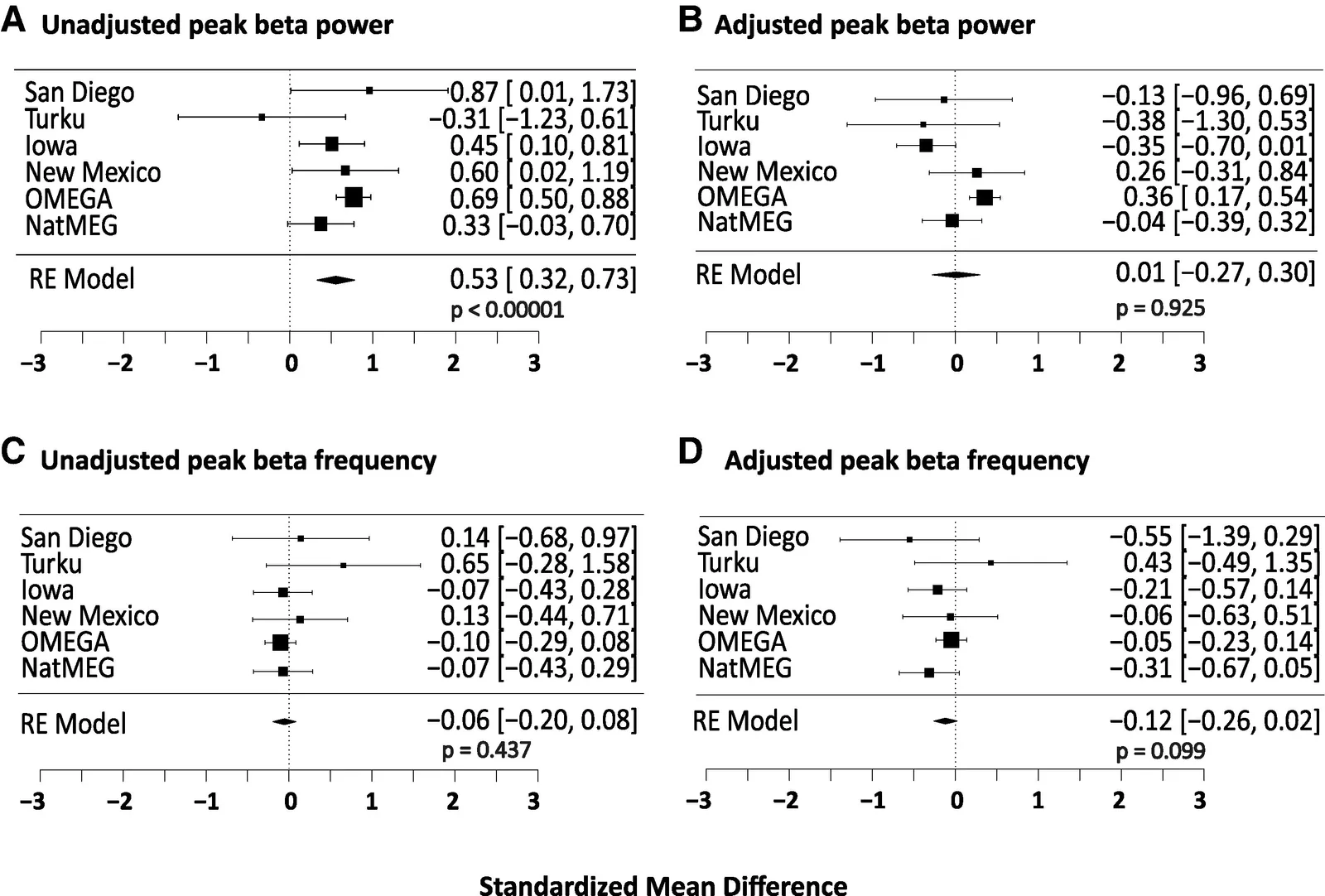
**Standardized mean differences in peak beta power and peak beta frequency between Parkinson’s patients (*N* = 368) and healthy controls (*N* = 570).** (**A**) Unadjusted peak beta power. (**B**) Adjusted peak beta power. (**C**) Unadjusted peak beta frequency. (**D**) Adjusted peak beta frequency. A positive number indicates values are higher in patients relative to controls, and a negative number indicates higher in controls relative to patients. Effect sizes for each feature were estimated using random-effects (RE) models with 95% confidence intervals estimated using restricted maximum likelihood (REML).

There was an effect of group on unadjusted beta power, SMD = 0.53, 95% CI = [0.32, 0.73], *P* < 0.00001, with higher unadjusted beta power in Parkinson’s disease patients relative to controls (Fig. 5A) across the scalp. There was no evidence of heterogeneity across the six datasets for unadjusted beta power: *Q*(5) = 7.63, *P* = 0.17, tau^2^ = 0.01, *I*^2^ = 29%.

### Adjusted peak beta power

After subtracting the aperiodic activity from the unadjusted beta power, there was no between-group difference in adjusted beta power, SMD = 0.01, 95% CI = [−0.27, 0.30], *P* = 0.925 (Fig. 5B). There was significant evidence of heterogeneity across datasets: *Q*(5) = 15.18, *P* = 0.009, tau*^2^* = 0.06, *I^2^* = 61.98%, indicating that effect sizes differed substantially across the six included studies.

### Unadjusted peak beta frequency

We identified no difference between Parkinson’s disease patients and controls for beta central frequency, SMD = −0.06, 95% CI [−0.20, 0.08], *P* = 0.437 (Fig. 5C). There was no evidence of heterogeneity: *Q*(5) = 3.15, *P* = 0.63, tau*^2^* = 0.0, *I^2^* = 0.0%, indicating stable effect size estimates across the six included studies.

### Adjusted peak beta frequency

After subtracting the aperiodic signal from the original spectrum, there was no difference in aperiodic-adjusted beta peak frequency between the two groups, SMD = −0.12, 95% CI = [−0.26, 0.02], *P* = 0.099 (Fig. 5D). There was no evidence of heterogeneity: *Q*(5) = 4.38, *P* = 0.496, tau^2^ = 0.000, *I*^2^ = 0.0%, suggesting that effect sizes were consistent across the datasets.

### Region of interest analysis

To examine whether any region-specific differences were present in the periodic and aperiodic features, we re-ran the RE models separately for three regions of interest across the scalp (frontal, central and posterior sensors). The standardized mean difference estimates from these region-specific models were broadly consistent with those obtained from the whole-scalp analysis, indicating that the results did not differ as a function of electrode/sensor region. The only exception was that the exponent was not different between the Parkinson’s disease and control groups in the posterior region (see Supplementary Figs 2–10 for the forest plots of frontal, central and posterior regions).

### Associations between M/EEG features and age

Due to limited clinical information across the six datasets (e.g. UPDRS score, medication dose, disease duration), a moderator analysis was considered not feasible and would likely have resulted in low statistical power. As age was the only variable consistently reported for all patients, we conducted 10 separate linear mixed-effects models within the full Parkinson’s disease patient cohort to examine potential age-related associations with M/EEG features.

A negative effect of age was observed on adjusted beta power: *b* = −0.003, SE = 0.001, *Z* = −2.79, *P* = 0.005, 95% CI [−0.005, −0.001]. Conversely, no relationship was found between age and unadjusted beta power: *b* = 0.003, SE = 0.002, *Z* = 1.05, *P* = 0.295, 95% CI [−0.002, 0.007], unadjusted beta peak frequency: *b* = 0.004, SE = 0.03, *Z* = 0.13, *P* = 0.89, 95% CI [−0.055, 0.063] or adjusted beta peak frequency: *b* = −0.029, SE = 0.016, *Z* = −1.80, *P* = 0.071, 95% CI = [−0.06, 0.002].

Within the alpha band, age was positively associated with unadjusted alpha power: *b* = 0.012, SE = 0.003, *Z* = 3.56, *P* < 0.0001, 95% CI [0.006, 0.019], and negatively associated with unadjusted alpha peak frequency: *b* = −0.048, SE = 0.008, *Z* = −5.94, *P* < 0.0001, 95% CI [−0.063, −0.032], and adjusted alpha peak frequency *b* = −0.037, SD = 0.007, *Z* = −5.11, *P* < 0.0001, 95% CI [−0.051, −0.023]. However, age was not associated with adjusted alpha power: *b* = 0.002, SE = 0.002, *Z* = 1.04, *P* = 0.30, 95% CI [−0.002, 0.006].

Significant positive effects of age were observed for both the aperiodic exponent: *b* = 0.009, SE = 0.003, *Z* = 3.48, *P* < 0.0001, 95% CI [0.004, 0.014], and the aperiodic offset: *b* = 0.016, SE = 0.005, *Z* = 3.25, *P* = 0.001, 95% CI [0.006, 0.026].

## Discussion

We conducted an advanced reanalysis and meta-analysis of resting-state M/EEG datasets to compare periodic and aperiodic features in patients with Parkinson’s disease and age-matched HCs. Using a common analytical pipeline, aggregating multiple raw, open-access M/EEG datasets, we compared the power and peak frequency of the aperiodic-adjusted alpha and beta oscillations, and the aperiodic features, i.e. exponent and offset across the two groups. A total of 6 datasets were included (4 recorded using EEG and 2 using MEG), involving 938 participants (368 with Parkinson’s disease and 570 HCs). In summary, we identified higher aperiodic offset and exponent in Parkinson’s patients relative to controls. Additionally, we found a higher unadjusted beta power in Parkinson’s patients, but after subtracting the aperiodic features from the original spectrum, there were no between-group differences in adjusted beta power or beta peak frequency. We also identified higher unadjusted and adjusted alpha power and slower peak alpha frequency in this resting-state data.

### Higher aperiodic exponent and offset in Parkinson’s disease

Compared with HCs, Parkinson’s patients had a consistently larger aperiodic exponent and offset across the six datasets. The functional relevance of the M/EEG exponent has been variously interpreted as an index of the neural excitation–inhibition ratio,^71-73^ oscillatory slowing^29^ and oscillation damping.^74^ We also observed a large effect of group on the aperiodic offset, with Parkinson’s disease patients showing larger offset values than HCs. Taken together, these aperiodic changes could indicate alterations in excitation–inhibition balance and increased baseline neural activity, reflecting pathological changes in network dynamics associated with Parkinson’s disease. Regardless of the physiological interpretation, our results suggest that elevated aperiodic exponent and offset are likely to be robust and reproducible characteristics across Parkinson’s disease studies.^33,41,42,71,72,75-82^

Three of the included datasets (New Mexico, NatMEG and OMEGA) had previously been analysed using spectral parameterization. These studies reported elevated aperiodic offset and exponent values in Parkinson’s disease patients, which our meta-analysis confirms are reliable markers consistently found in Parkinson’s disease across the datasets. On a methodological note, although our analysis pipeline is broadly comparable, each study employed slightly different parameterization approaches, with minor variations in the parameters and frequency ranges. A systematic investigation of how these methodological choices influence the spectral parameterization outputs, and the establishment of best-practice guidelines would improve consistency across research groups. Reliable measures are important for meaningful comparisons across individuals and groups over time, indicating that observed changes reflect biological factors rather than measurement errors.^83,84^ Both aperiodic offset and exponent show good test–retest reliability,^85,86^ indicating that these features are stable over time and capable of capturing individual differences in neural activity. This reliability strengthens their potential as markers for investigating neural mechanisms and clinical conditions.

Whilst our results and previous studies^18,33^ provide evidence that the aperiodic offset can distinguish Parkinson’s disease patients from HCs, its functional relevance remains unclear. Although the aperiodic offset and exponent have been associated with motor symptom severity in Parkinson’s disease,^77^ conflicting evidence has also been reported.^80^ Similarly, cognitive decline in Parkinson’s disease has also been associated with aperiodic activity in some studies,^29,79^ but this association could not be replicated in others.^53^ Overall, it remains uncertain whether the aperiodic offset and exponent are directly linked to cognitive and motor symptoms in Parkinson’s disease.

### Physiological interpretation of aperiodic changes

Recent advancements in electrophysiological signal analysis have made it possible to detect changes in the excitation–inhibition ratio, which may be detectable during (and potentially in the prodrome of) disease processes. A link between the aperiodic exponent and the E/I ratio in the brain has been reported.^37,38^ Ketamine, by blocking NMDA receptor-mediated input to inhibitory interneurons, induces cortical disinhibition and increases glutamate release. In contrast, thiopental, a GABA_A_ receptor modulator, suppresses cortical excitation.^87^ Both drugs have been shown to modulate the aperiodic exponent in healthy individuals.^88^ However, other pharmacological manipulations of inhibitory neural mechanisms have failed to support the hypothesis that the aperiodic exponent reliably reflects the E/I ratio in electrophysiological activity.^89^ Although the relationship between the aperiodic exponent and E/I balance is debated, these findings highlight the broader importance of modelling aperiodic activity when interpreting electrophysiological changes in Parkinson’s disease.

Both invasive and non-invasive electrophysiology in humans and animals have identified elevated baseline neuronal activity in Parkinson’s disease, reflected in M/EEG as a higher aperiodic offset. Alterations in the balance of excitation and inhibition have also been reported, consistent with a larger aperiodic exponent (i.e. a steeper spectral slope).^41,71,73,79,90,91^ Increases in both the aperiodic exponent and offset in Parkinson’s disease patients relative to controls have been documented.^29,33^ However, several studies have reported either a reversed pattern^76,92,93^ or no group-level differences in these aperiodic parameters.^53,75,77,78,94^ Whether the aperiodic exponent can be adopted as a marker of E/I balance remains a matter of debate. The aperiodic exponent is not a one-to-one measurement of inhibition or excitation; e.g. increased inhibition may increase the aperiodic exponent, whereas increased excitation may not necessarily lead to a corresponding decrease.^89^ Therefore, multiple physiological mechanisms can produce similar changes in aperiodic activity, and alternative interpretations remain possible. Although there is growing interest in aperiodic activity as a potential correlate of disease progression, recent work emphasizes that it should not be treated as a direct or exclusive index of E/I balance.^91^

### No group-level differences in resting-state beta activity after removal of the aperiodic exponent

One of our key findings is the divergence between adjusted and aperiodic-adjusted beta power. We identified no difference in beta power between individuals with Parkinson’s disease and HCs when we extracted the aperiodic exponent from the signal. In contrast, and in line with much of the previous literature, Parkinson’s patients had a higher beta power compared with controls when we performed a meta-analysis of the unadjusted signal (i.e. without the removal of the exponent). These findings suggest that previously reported group differences in beta power may, at least in part, reflect contamination by aperiodic activity rather than true oscillatory beta-band alterations, raising the possibility that genuine resting-state beta differences between Parkinson’s patients and controls are smaller or absent when properly accounting for aperiodic features of the signal.

While excess beta activity is a known hallmark of Parkinson’s disease, the supporting evidence mostly comes from intracranial subthalamic recordings. In this meta-analysis of M/EEG activity, we found no differences in aperiodic-adjusted cortical beta power or beta peak frequency between individuals with Parkinson's disease and HCs. Although we analysed cortical activity at resting-state, and beta-band activity (13–30 Hz) is more strongly associated with motor preparation and execution,^95,96^ there is a large body of evidence showing elevated subthalamic beta activity in Parkinson’s disease patients,^14,16,80,97-101^ particularly at rest.^102^ Additionally, medication and DBS treatment have been shown to improve motor symptoms while suppressing subthalamic beta activity.^100,102^ Moreover, it has been shown that cortical and subcortical beta activity are covariant.^103^ However, our results suggest that averaged cortical rest-state beta power by itself may not serve as a reliable correlate of Parkinson’s disease, given the inconsistencies observed between unadjusted and adjusted beta-power measures.

Our decision to analyse peak power within canonical frequency bands may not have been sensitive enough to capture important pathological electrophysiological changes in Parkinson’s disease. Beta activity often occurs in transient bursts with varying duration and amplitude.^8,16,104^ Notably, beta bursts occurring close in time to motor movements are associated with slower response times,^105^ and neurofeedback-driven suppression of these bursts leads to faster reaction times.^104^ Thus, analysing the temporal dynamics of beta bursts may offer a more sensitive and reliable method for investigating cortical motor impairments in Parkinson’s disease.^106^

### Higher peak alpha power and slower peak alpha frequency in Parkinson’s disease

The meta-analyses of alpha band measures were broadly consistent across the six included datasets, with statistical indices suggesting low between-study heterogeneity for both higher peak alpha power and slower peak alpha frequency in Parkinson’s disease patients relative to controls.^24,30,107,108^ However, the relevance of this change in the clinical population is not immediately clear. In healthy adults, alpha rhythms are proposed to reflect decreased excitability of the underlying cortex and are modulated by both tonic and selective attention.^22,23^ For example, increases in alpha power are observed during fatigued states,^109,110^ and linear increases in alpha activity are commonly recorded during the course of experimental sessions, as participants experience fatigue as a result of extended engagement with perceptual tasks (the ‘time-on-task’ effect).^111,112^

Similarly, Parkinson’s disease presents not only with motor symptoms but also with a range of non-motor symptoms, such as fatigue and apathy.^113-118^ Around half of Parkinson’s disease patients experience fatigue, both in terms of reduced physical and mental energy and increased effort in cognition and perception, at higher levels than healthy individuals.^118^ Apathy is characterized by diminished motivation, goal-directed behaviour and emotional engagement, manifesting as indifference, lack of initiative and impaired decision-making.^117,119^ Both apathy and fatigue can appear early in Parkinson’s disease, often preceding motor symptoms, and their prevalence often increases as the disease progresses.^118-120^

Although we identified a consistent increase in alpha power in patients relative to controls, which may be related to transient or chronic episodes of fatigue and apathy, none of the datasets included in the meta-analyses included clinical fatigue measurements to test this hypothesis directly. The OMEGA dataset did include scores from the Unified Parkinson's Disease Rating Scale Part I (UPDRS-I), which contains subitem 13 in UPDRS-I, assessing fatigue over the past few weeks. However, these scores were unavailable in other datasets, precluding a formal meta-regression. Furthermore, although subitem 13 in UPDRS-I assesses fatigue, it may not serve as a reliable indicator of the fatigue state experienced at the time of M/EEG recording. As such, we cannot determine whether the observed alterations in alpha oscillations are driven by underlying pathology, cognitive changes, elevated apathy or fatigue levels, or a combination of these factors. We recommend that future research includes both physical and cognitive fatigue measures taken at the time of recording, alongside longer-term assessments, to better understand the relationship between Parkinson’s disease and increased alpha power.

In addition to increased alpha power, we also identified a slowing of peak alpha frequency in Parkinson’s patients relative to controls. Although subtle, this effect was consistent across datasets, with a mean slowing of 0.92 Hz (SD = 0.062). Individual alpha frequency (IAF) gradually changes non-linearly throughout the lifespan,^121-131^ typically peaking around 10 Hz on average in young adulthood,^129^ followed by a gradual slowing into older age. The functional significance of IAF is still under debate (see^132^ for a review) but has been proposed to represent the ‘sampling rate’ of the perceptual system,^133^ suggesting that slowing of IAF in Parkinson’s patients may reflect disrupted perceptual or cognitive processing.^134^ Specifically, Parkinson’s disease patients with more pronounced alpha slowing show increased disease progression and cognitive decline in longitudinal studies.^19^ These findings indicate that pathological changes in neural competence in Parkinson’s disease may directly impact neural oscillations. However, these changes could also reflect secondary effects of non-motor symptoms commonly observed in Parkinson’s disease.

Aperiodic-adjusted alpha power had previously been reported for three of the six included datasets (OMEGA, NatMEG and New Mexico). Our reanalysis using a single common processing pipeline led to similar conclusions as these studies in the alpha band; all three studies had previously reported an increased alpha power in Parkinson’s disease patients relative to controls and we replicated this effect here. Of the six datasets included in our analyses, only one (NatMEG) had previously examined peak alpha frequency between groups and had reported no differences. In contrast, our reanalysis of the raw data identified a significant slowing of peak alpha frequency in Parkinson’s disease relative to controls in the NatMEG dataset (and consistently across all datasets). This discrepancy could be due to differences in the selection of sensors analysed; the original analysis focused on a frontocentral sensorimotor region of interest,^33^ whereas our approach used full-scalp data, including the posterior sensors.

### Limitations and future directions

In our reanalysis of existing raw M/EEG datasets, we developed a common processing pipeline that was applied to all six datasets to ensure that the periodic and aperiodic features were extracted from consistently cleaned and processed signals. As shown in Supplementary Fig. 1, varying the frequency ranges used for spectral parameterization substantially affects the outputs, particularly the estimate of the aperiodic exponent. For example, parameterizing the spectrum from 1 to 20 Hz results in a smaller exponent compared with using 3–40 Hz. In addition, identifying peaks within the canonical frequency bands involves researcher-led definitions of the upper and lower limits of those bands of interest (e.g. alpha may be variously defined as 8–12 Hz or 8–13 Hz, or further subdivided into low (8–10 Hz) and high (10–12 Hz) alpha). Although both the clinical and control groups in this study were processed using the same pipeline, allowing for a comparison of standardized mean differences between the two groups, the impact of these experimenter-defined choices on the outputs of spectral parameterization is underexplored.

Second, to facilitate data synthesis and ensure comparability across datasets, we chose to focus our main analysis on scalp-wide averages of the data across all available sensors. In response to a suggestion put forward during peer review, we performed three further sets of analyses, separately for the frontal, central and posterior scalp regions, but we did not identify systematic differences for any of the M/EEG features, except that the exponent did not differ between patients and controls in the posterior region.

It is also important to note that our analyses were based on a combination of eyes-open (Iowa, San Diego, New Mexico, Turku and OMEGA) and eyes-closed (NatMEG) resting-state recordings. This may limit the generalizability of our findings to M/EEG collected during other paradigms or active task states, particularly for alpha-band measures. However, across all eight M/EEG features examined, the standardized mean differences between patients and controls did not identify the eyes-closed NatMEG dataset as an outlier relative to the others. Nonetheless, the inclusion of five eyes-open datasets and one eyes-closed dataset to maximize sample size should be considered when interpreting our results in the context of the wider literature.

Our decision to focus the analysis on the alpha and beta frequency windows was guided by the peaks observed in the grand-average spectral plots (Fig. 2). Consequently, we did not perform a synthesis of other frequency bands, such as the theta band, which has been reported to change in Parkinson’s disease and may be a correlate of Parkinson’s disease-related cognitive decline.^24,135,136^ We also chose to only include Parkinson’s disease patients who were tested ON medication, due to the low availability of both ON- and OFF-medication datasets. It remains possible that different effects, particularly in beta power and aperiodic features, would emerge when comparing patients tested in OFF-medication states to controls. Further research examining ON versus OFF medication states, as well as recording during active movement, is needed to evaluate the reliability of spectral parameterization as a correlate of Parkinson’s disease. Additionally, it remains unclear whether the periodic and aperiodic changes observed in Parkinson’s disease represent specific disease biomarkers or simply correlate with disease presence.

## Conclusion

Parkinson’s patients showed consistently elevated aperiodic exponent and offset, indicating robust broadband differences in resting-state M/EEG activity. Differences in beta activity were only present for unadjusted beta power, with the effects not present in the aperiodic-adjusted analysis, suggesting that previously reported beta abnormalities may reflect aperiodic contributions rather than true oscillatory changes. Finally, patients exhibited higher unadjusted alpha power and a slower unadjusted peak alpha frequency, with both effects remaining present, although reduced, after aperiodic adjustment. This meta-analysis highlights the importance of removing aperiodic signal from M/EEG data when assessing oscillatory correlates of clinical conditions. Further research is needed to refine these electrophysiological measures (particularly with best-practice guidelines for parameter selection during the processing stages), and to explore their potential clinical applications in the diagnosis and treatment of neurodegenerative conditions.

## Supplementary Material

fcag329_Supplementary_Data

## Data Availability

The full analysis scripts are available at https://github.com/HamzehNorouzi/Meta_Reanalysis. The data for five datasets were publicly available from the following locations: New Mexico (https://openneuro.org/datasets/ds003490/versions/1.1.0), Iowa (https://openneuro.org/datasets/ds004584/versions/1.0.0), Turku (https://osf.io/pehj9/), San Diego (https://openneuro.org/datasets/ds002778/versions/1.0.2) and NatMEG (https://search.kg.ebrains.eu/instances/d55146e8-fc86-44dd-95db-7191fdca7f30). The data for the OMEGA dataset were obtained directly from the original study authors upon request.
