# Supplementary material for: A meta-analysis of periodic and aperiodic electrophysiological features in Parkinson’s disease
Source: Brain Commun. 2026 Aug 28;8(5):fcag329. doi: 10.1093/braincomms/fcag329 (PMC13559478; doi:10.1093/braincomms/fcag329)
Supplement: Supplementary file 1 [file fcag329_supplementary_data.pdf]

## Supplementary Materials

A recent study [1] reported that approximately 50% of previous research decomposing periodic and aperiodic components and estimated exponent values used a frequency range below 43 Hz. Furthermore, 85% of studies employing frequency ranges below 40 Hz selected ranges with a minimum frequency of 3 Hz. While some research recommends conducting spectral parameterisation within a narrow frequency window, others argue that broader frequency windows improve model performance and affect key parameters such as the exponent and offset [2]. To systematically assess these effects, we tested multiple window sizes during the development of our spectral parameterisation pipeline, specifically: 1–20 Hz, 1–30 Hz, 1–40 Hz, 1–50 Hz, 1–60 Hz, 1–70 Hz, 1–80 Hz, 1–90 Hz, 3–20 Hz, 3–30 Hz, 3–40 Hz, 3–50 Hz, 3–60 Hz, 3–70 Hz, 3–80 Hz, 3–90 Hz). The results are shown in Supplementary Figure 1 below.

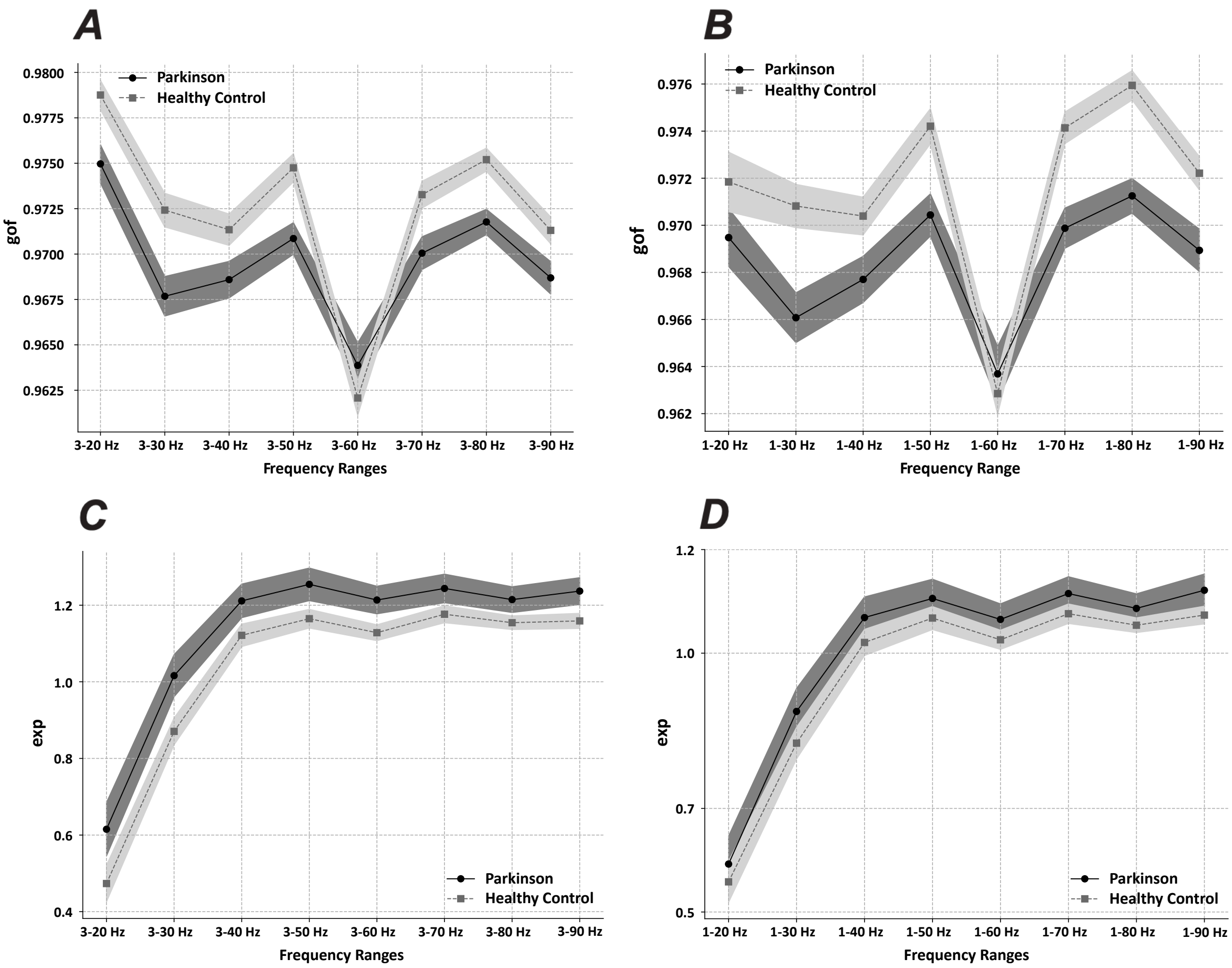


**Supplementary Figure 1. Effects of different frequency ranges on Specparam model variables across all 6 datasets and all participants (Parkinson's patients: N = 368, healthy controls: N = 570)**. A) Goodness of fit (gof) values for frequency windows of 3-F Hz. B) Model goodness of fit values for frequency windows of 1-F Hz. C) Exponent values for frequency windows of 3-F Hz. D) Exponent values for frequency windows of 1-F Hz. Each point and shaded area represent the mean and 95% confidence intervals of the variable. gof = Model goodness of fit, exp = Exponent.

## Supplementary Materials

Supplementary Figures 1A and 1B illustrate the model goodness of fit across different frequency ranges. A slight decrease in model goodness of fit was observed in the ranges of 1-60 Hz and 3-60 Hz due to power line noise. We also found that frequency ranges 1–20 Hz, 3–20 Hz, 1–30 Hz, and 3–30 Hz did not provide stable exponent values, with smaller windows generating shallower slopes, although exponent values plateaued in broader windows over 40 Hz (Supplementary Figures 1C and 1D). Based on these observations, and to maintain consistency with prior research, we selected the 3–40 Hz range for our analysis.

Supplementary Materials

Supplementary Table 1. Mean and standard deviation of targeted features for PD patients.

| DATASET | PD exponent | PD offset | PD unadjusted alpha-amplitude | PD adjusted alpha-amplitude | PD unadjusted alpha-Peak Frequency | PD adjusted alpha-Peak frequency | PD unadjusted beta-amplitude | PD adjusted beta-amplitude | PD unadjusted beta-Peak Frequency | PD adjusted beta-Peak Frequency |
|---|---|---|---|---|---|---|---|---|---|---|
| SanDiego (13 PD[U]) | 1.24 (0.14) | -11.573 (0.241) | -12.09(.0.48) | 0.605 (0.341) | 9.11 (1.125) | 8.4 (1.04) | -12.64 (0.309) | 0.398 (0.122) | 19.804 (3.539) | 15.19 (1.812) |
| Turku (6 PD[U]) | 1.236 (0.16) | -11.34 (0.222) | -12.16(0.691) | 0.522 (0.218) | 8.567 (1.547) | 7.76 (1.30) | -12.47 (0.55) | 0.261 (0.3) | 19.45 (3.797) | 16.19 (3.745) |
| Iowa (115 PD[U]) | 1.036 (0.341) | -11.59 (0.585) | -11.77(0.536) | 0.754 (0.294) | 8.652 (1.506) | 8.44 (1.29) | -12.52 (0.4) | 0.3 (0.184) | 17.403 (3.556) | 15.48 (2.76) |
| NewMexico (24 PD[U]) | 1.138 (0.236) | -11.56 (0.464) | -11.9(0.511) | 0.746 (0.221) | 9.367 (1.536) | 9.21 (1.46) | -12.49 (0.32) | 0.435 (0.166) | 18.05 (3.017) | 15.79 (3.04) |
| OMEGA (154 PD[U]) | 1.373 (0.518) | -25.81 (0.9) | -26.39(0.645) | 0.701 (0.265) | 9.13 (1.369) | 8.49 (1.417) | -26.99 (0.48) | 0.475 (0.193) | 18.503 (3.7) | 15.39 (2.63) |
| NatMEG (56 PD[U]) | 1.148 (0.254) | -27.14 (0.341) | -27.48(0.33) | 0.735 (0.253) | 9.036 (1.175) | 8.65 (1.109) | -28.04 (0.22) | 0.453 (0.147) | 16.542 (3.784) | 14.88 (1.68) |
| **Mean (SD)** | **EEG: 1.16 (0.22), MEG: 1.26 (0.38)** | **EEG: -11.516 (0.378), MEG: -26.475 (0.67)** | **EEG: -11.98 (0.556), MEG = -26.93 (0.487)** | **EEG: 0.66 (0.26), MEG: 0.71 (0.26)** | **EEG: 8.92 (1.42), MEG: 9.08 (1.27)** | **EEG: 8.45 (1.27), MEG: 8.57 (1.26)** | **EEG: -12.59 (0.38), MEG: --27.51 (0.35)** | **EEG: 0.35 (0.19), MEG: 0.46 (0.17)** | **EEG: 18.67 (3.47), MEG: 17.52 (3.74)** | **EEG: 15.66 (2.84), MEG: 15.13 (2.15)** |

Supplementary Table 2. Mean and standard deviation of targeted features for healthy controls.

| DATASET | HC exponent | HC offset | HC unadjusted alpha-amplitude | HC adjusted alpha-amplitude | HC unadjusted alpha-Peak Frequency | HC adjusted alpha-Peak Frequency | HC unadjusted beta-amplitude | HC adjusted beta-amplitude | HC unadjusted beta-Peak Frequency | HC adjusted beta-Peak Frequency |
|---|---|---|---|---|---|---|---|---|---|---|
| SanDiego (10 HC[a]) | 1.197 (0.117) | -11.862 (0.31) | -12.41 (0.48) | 0.609 (0.364) | 9.845 (1.186) | 9.53 (1.41) | -12.92 (0.313) | 0.419 (0.182) | 19.275 (3.669) | 16.54 (2.92) |
| Turku (42 HC[a]) | 1.02 (0.187) | -11.663 (0.918) | -12.16 (0.96) | 0.454 (0.328) | 10.006 (1.279) | 9.04 (1.36) | -12.47 (0.9) | 0.35 (0.2) | 18.247 (0.471) | 15.2 (1.62) |
| Iowa (42 HC[a]) | 0.937 (0.258) | -11.948 (0.465) | -12.21 (0.60) | 0.575 (0.364) | 9.94 (1.298) | 9.01 (1.14) | -12.68 (0.37) | 0.366 (0.201) | 17.657 (3.041) | 16.06 (2.47) |
| NewMexico (23 HC[a]) | 0.943 (0.209) | -11.907 (0.37) | -12.27 (0.54) | 0.542 (0.281) | 9.639 (1.802) | 9 (1.16) | -12.7 (0.36) | 0.393 (0.145) | 17.634 (3.103) | 15.96 (2.45) |
| OMEGA (411 HC[a]) | 1.183 (0.379) | -26.249 (0.804) | -26.78 (0.56) | 0.639 (0.283) | 10.206 (1.24) | 9.64 (1.41) | -27.28 (0.39) | 0.409 (0.182) | 18.909 (3.957) | 15.51 (2.43) |
| NatMEG (64 HC[a]) | 0.932 (0.187) | -27.48 (0.298) | -27.69 (0.41) | 0.66 (0.288) | 9.75 (1.08) | 9.34 (1.14) | -28.13 (0.3) | 0.459 (0.18) | 16.79 (3.025) | 15.48 (2.08) |
| **Mean (SD)** | **EEG: 1.02 (0.19),MEG: 1.05 (0.28)** | **EEG: -11.845 (0.516), MEG = -26.865 (0.551)** | **EEG: -12.26 (0.64), MEG: -27.23 (0.48)** | **EEG: 0.54 (0.33), MEG: 0.64 (0.28)** | **EEG: 9.85 (1.39), MEG: 9.97 (1.16)** | **EEG: 9.14 (1.27), MEG: 9.49 (1.28)** | **EEG: -12.69 (0.48), MEG: -27.7 (0.34)** | **EEG: 0.38 (0.18), MEG: 0.43 (0.18)** | **EEG: 18.2 (2.57), MEG: 17.84 (3.49)** | **EEG: 15.94 (2.37), MEG: 15.49 (2.25)** |

## Supplementary Materials

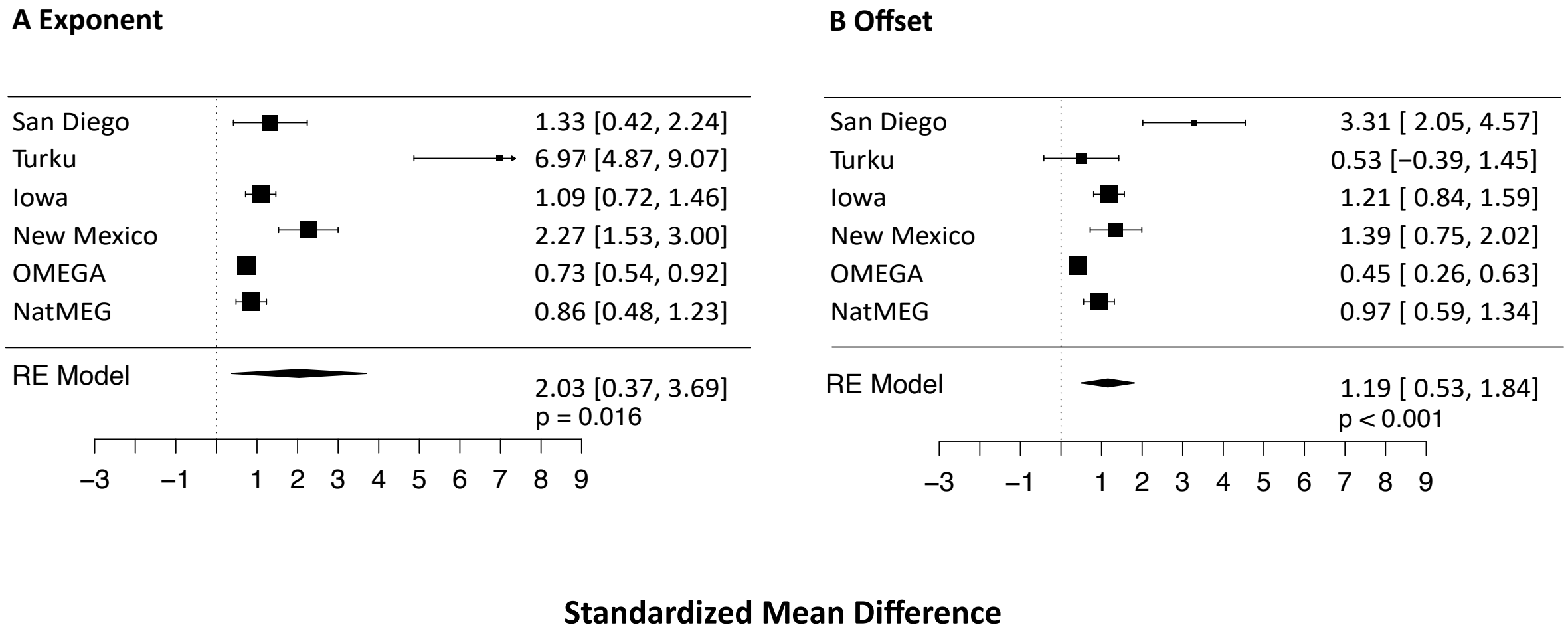


**Supplementary Figure 2. Forest plots for aperiodic components across frontal sensors.** Effect sizes for each feature were estimated using random effects (RE) models with 95% confidence intervals estimated using restricted maximum likelihood (REML).

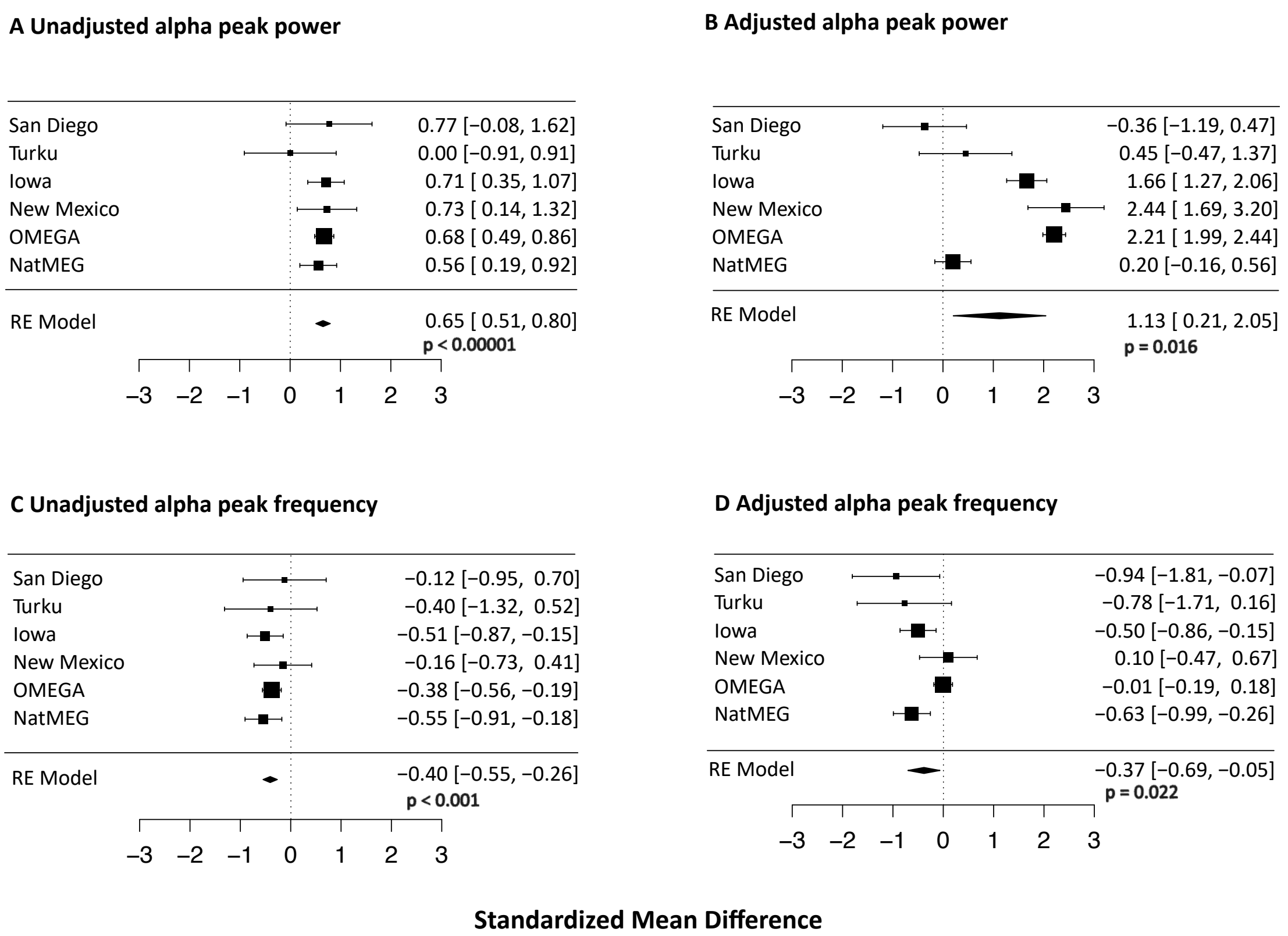


**Supplementary Figure 3. Forest plots for peak alpha power and peak alpha frequency across frontal sensors.** Effect sizes for each feature were estimated using random effects (RE) models with 95% confidence intervals estimated using restricted maximum likelihood (REML).

## Supplementary Materials

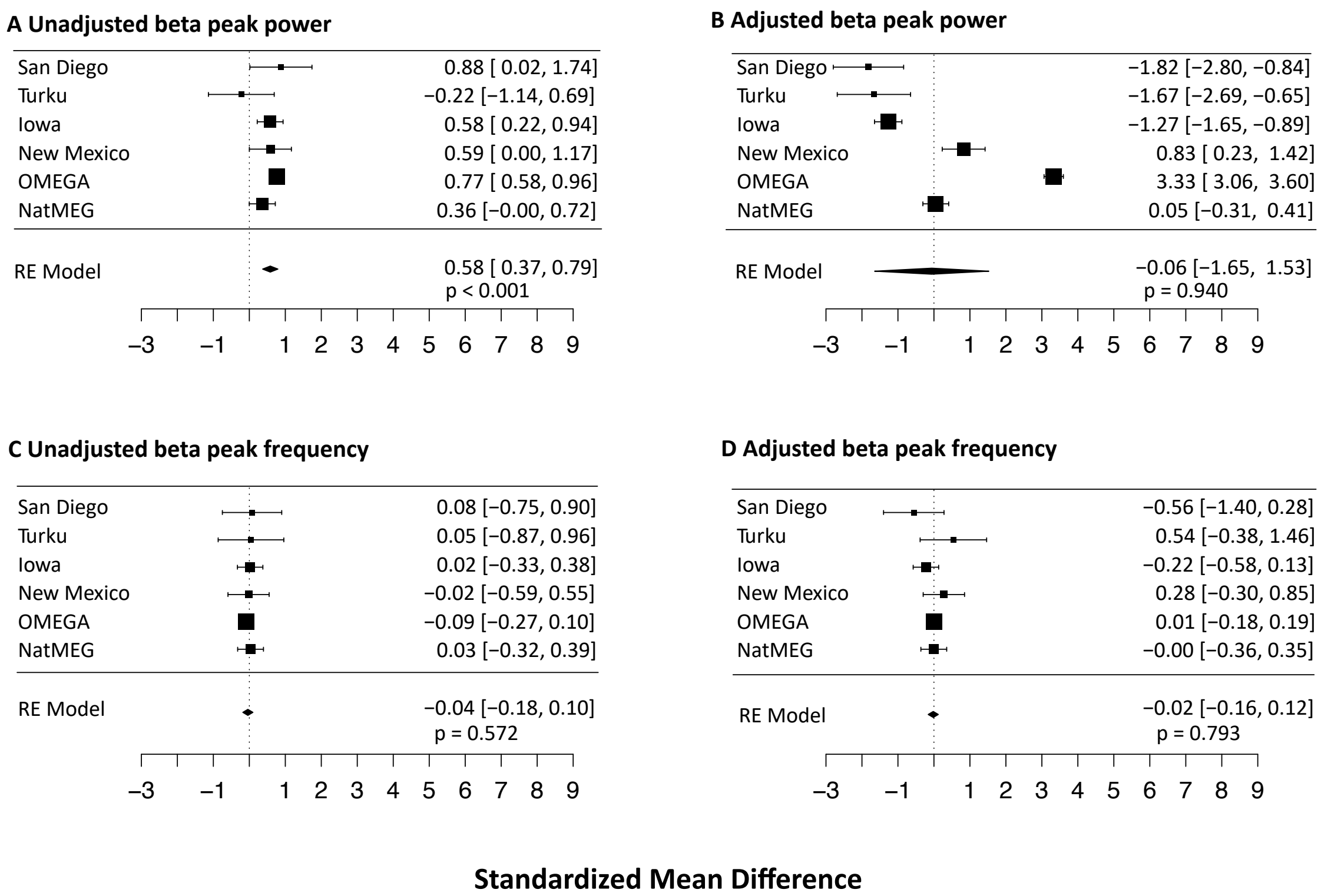


**Supplementary Figure 4. Forest plots for beta peak power and peak frequency across frontal sensors.** Effect sizes for each feature were estimated using random effects (RE) models with 95% confidence intervals estimated using restricted maximum likelihood (REML).

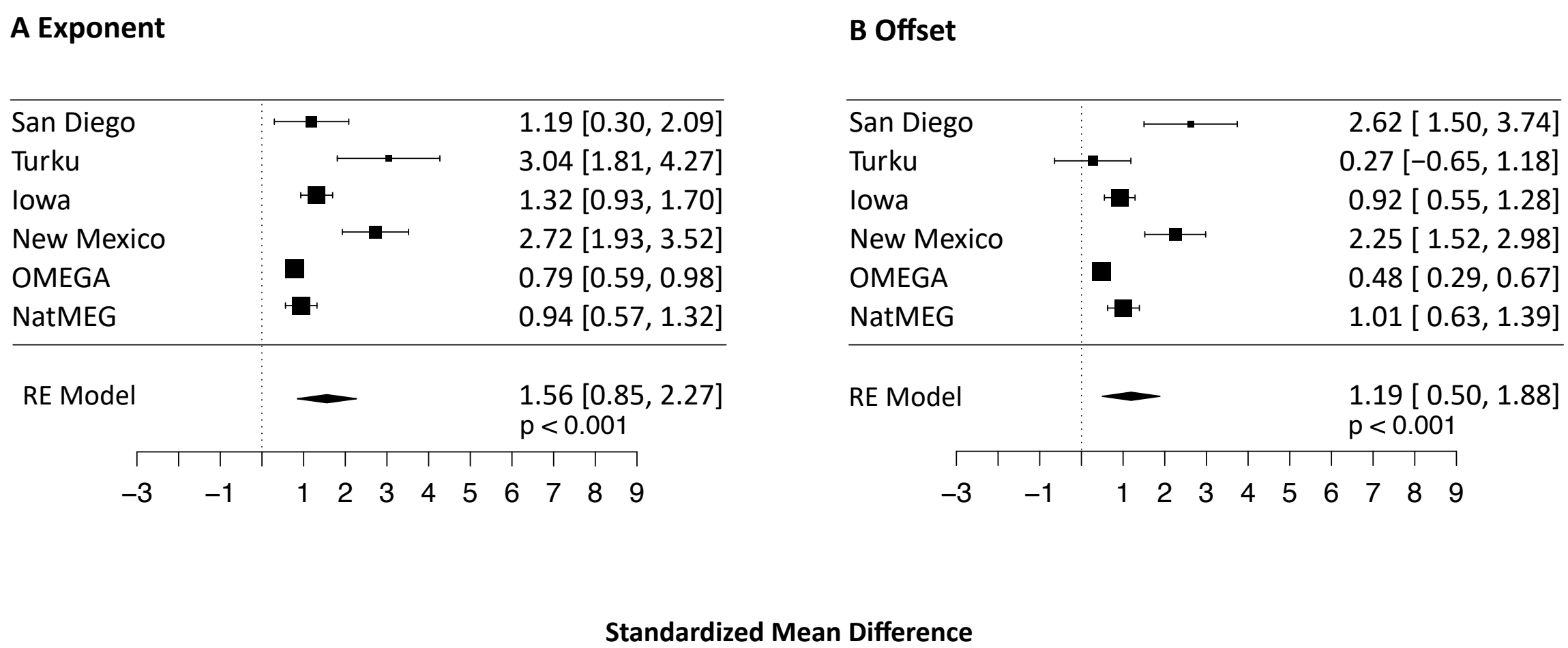


**Supplementary Figure 5. Forest plots for aperiodic components across central sensors.** Effect sizes for each feature were estimated using random effects (RE) models with 95% confidence intervals estimated using restricted maximum likelihood (REML).

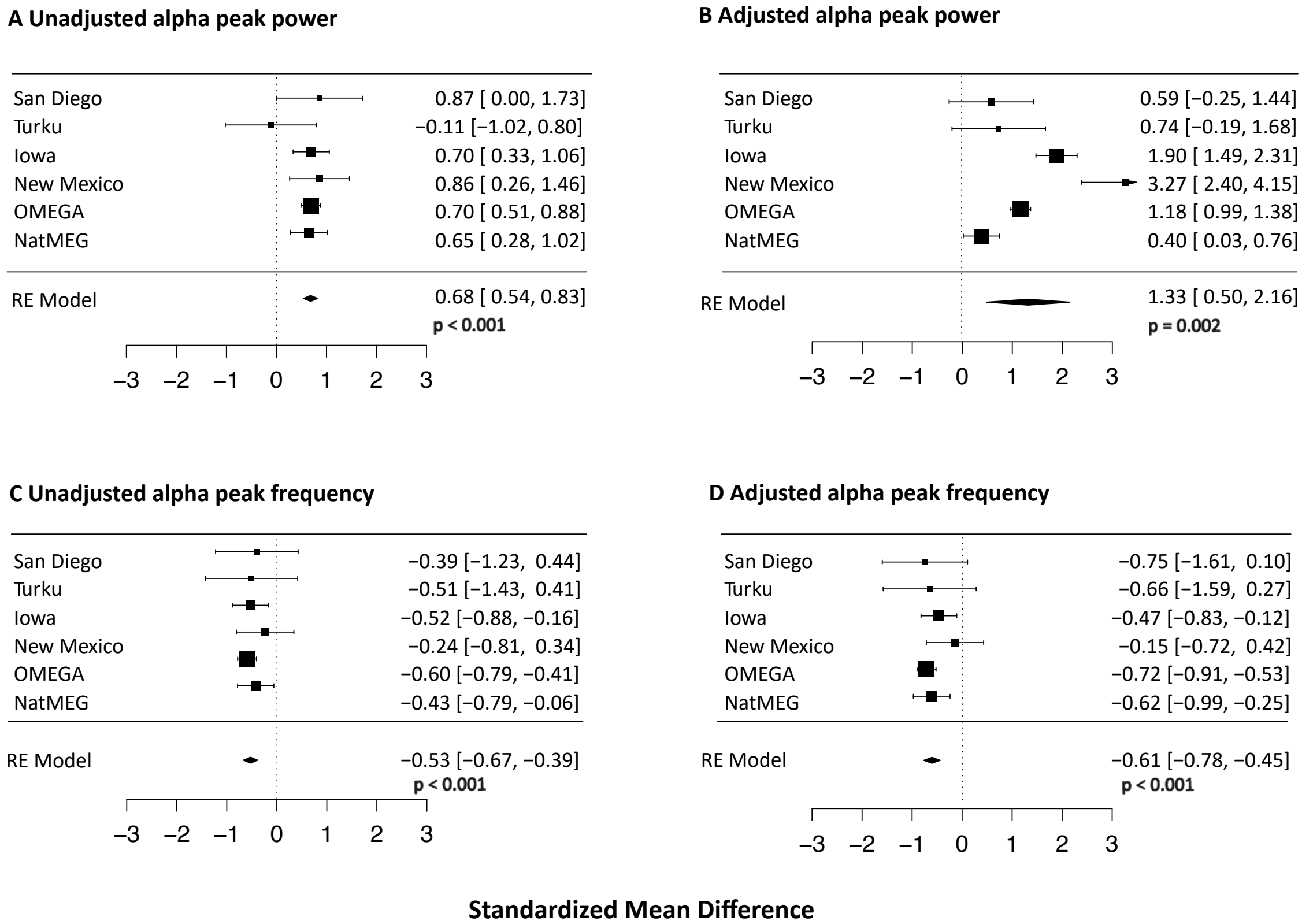


**Supplementary Figure 6. Forest plots for alpha peak power and peak frequency across central sensors.** Effect sizes for each feature were estimated using random effects (RE) models with 95% confidence intervals estimated using restricted maximum likelihood (REML).

## Supplementary Materials

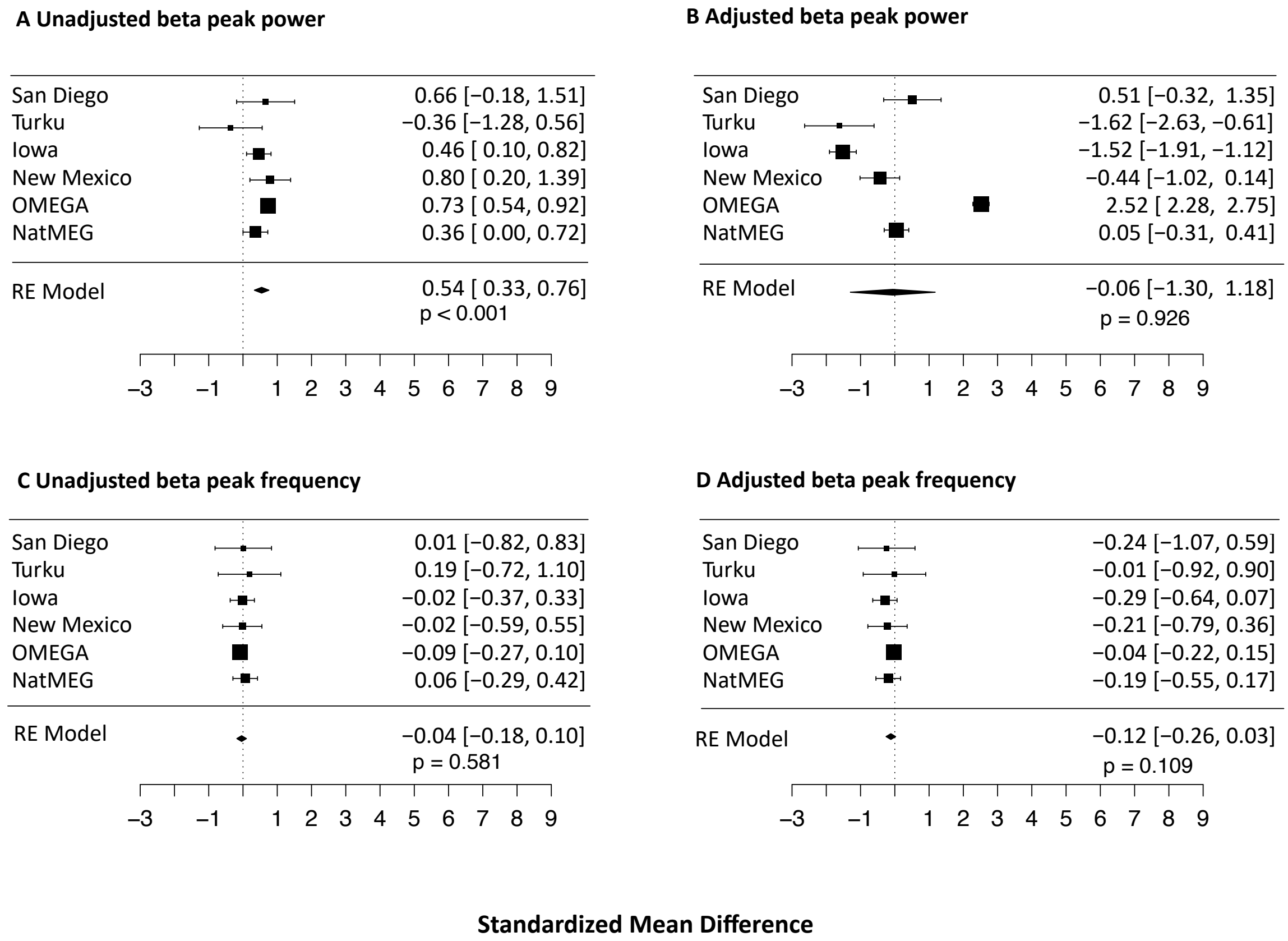


**Supplementary Figure 7. Forest plots for beta peak power and peak frequency across central sensors.** Effect sizes for each feature were estimated using random effects (RE) models with 95% confidence intervals estimated using restricted maximum likelihood (REML).

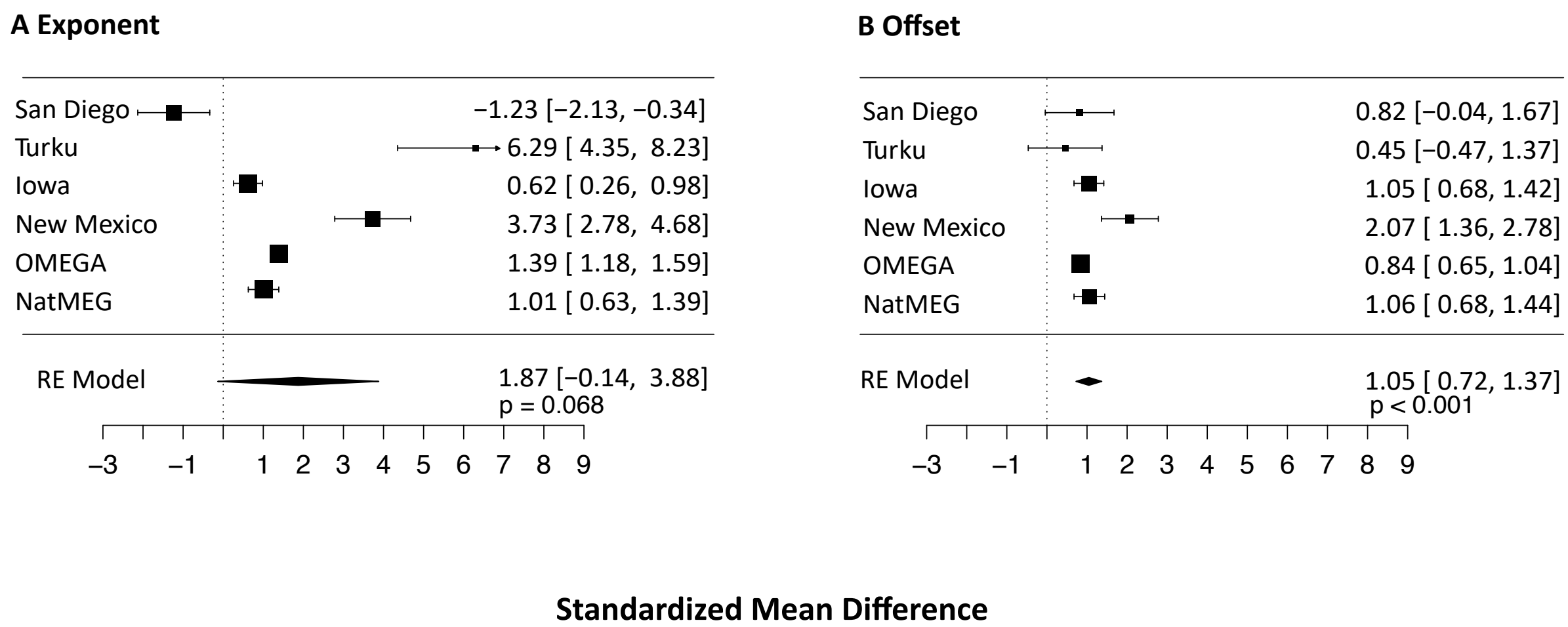


**Supplementary Figure 8. Forest plots for aperiodic components across posterior sensors.** Effect sizes for each feature were estimated using random effects (RE) models with 95% confidence intervals estimated using restricted maximum likelihood (REML).

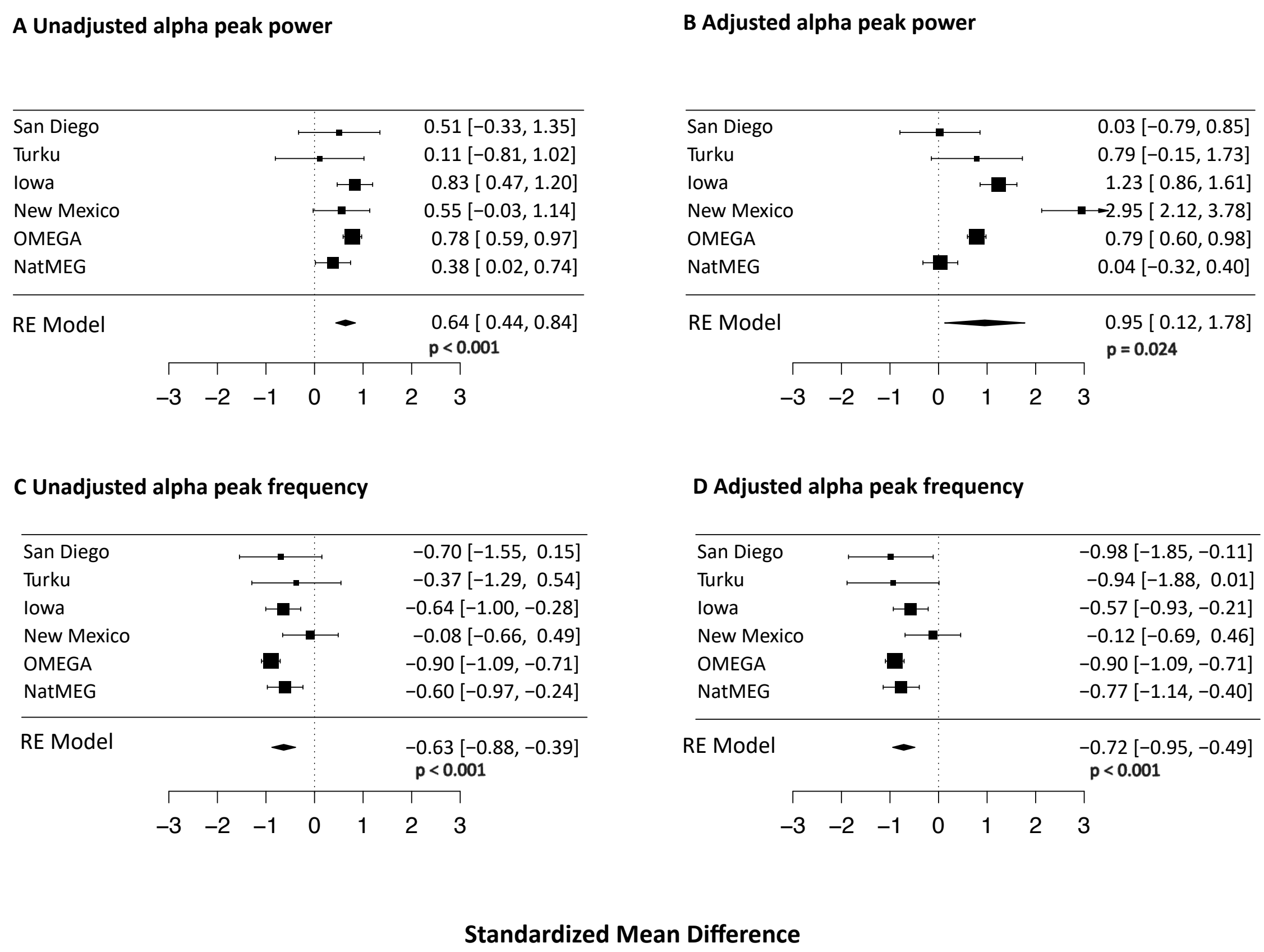


**Supplementary Figure 9. Forest plots for peak alpha power and peak alpha frequency across posterior sensors.** Effect sizes for each feature were estimated using random effects (RE) models with 95% confidence intervals estimated using restricted maximum likelihood (REML).

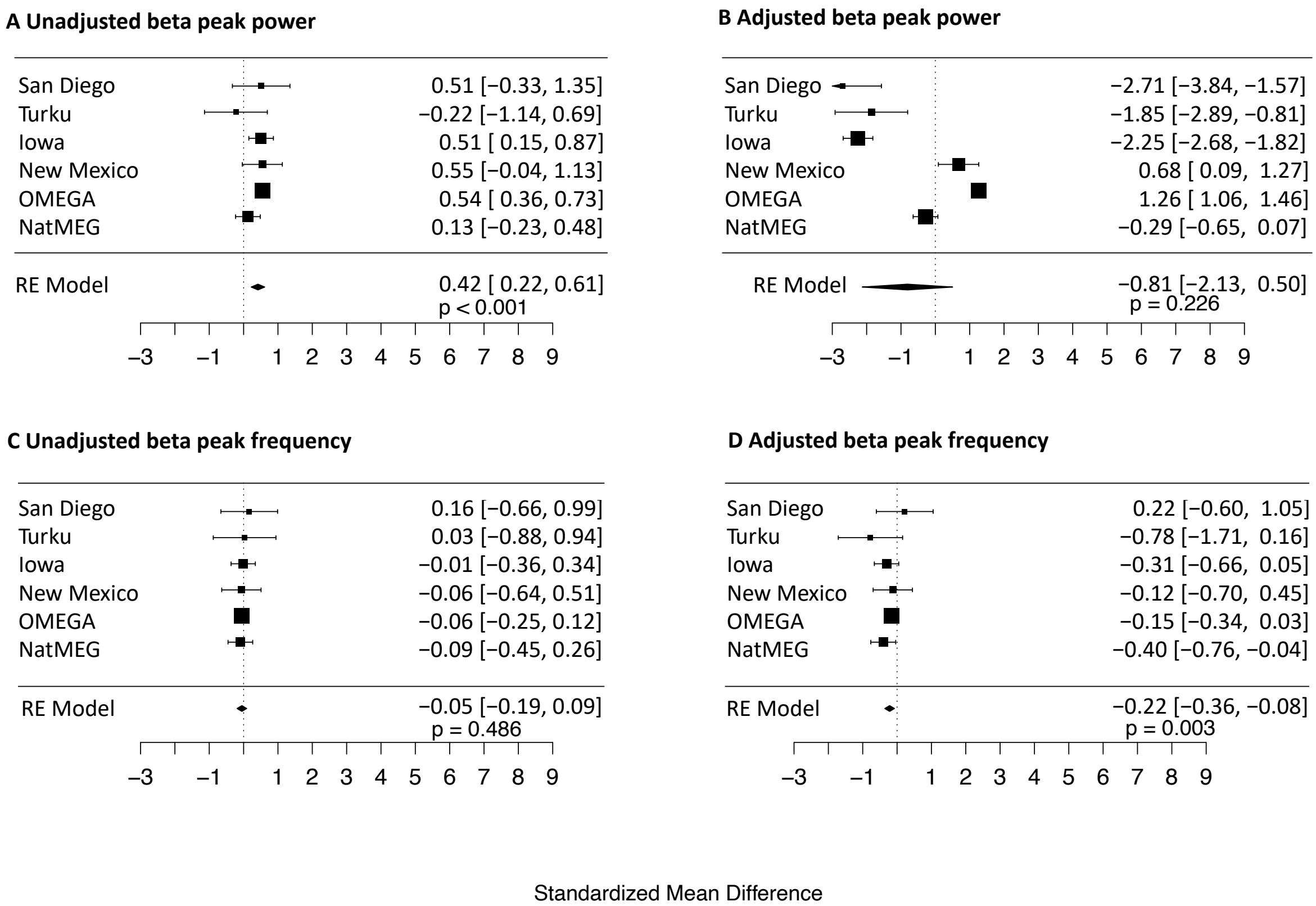


**Supplementary Figure 10. Forest plots for beta peak power and peak frequency across posterior sensors.** Effect sizes for each feature were estimated using random effects (RE) models with 95% confidence intervals estimated using restricted maximum likelihood (REML).

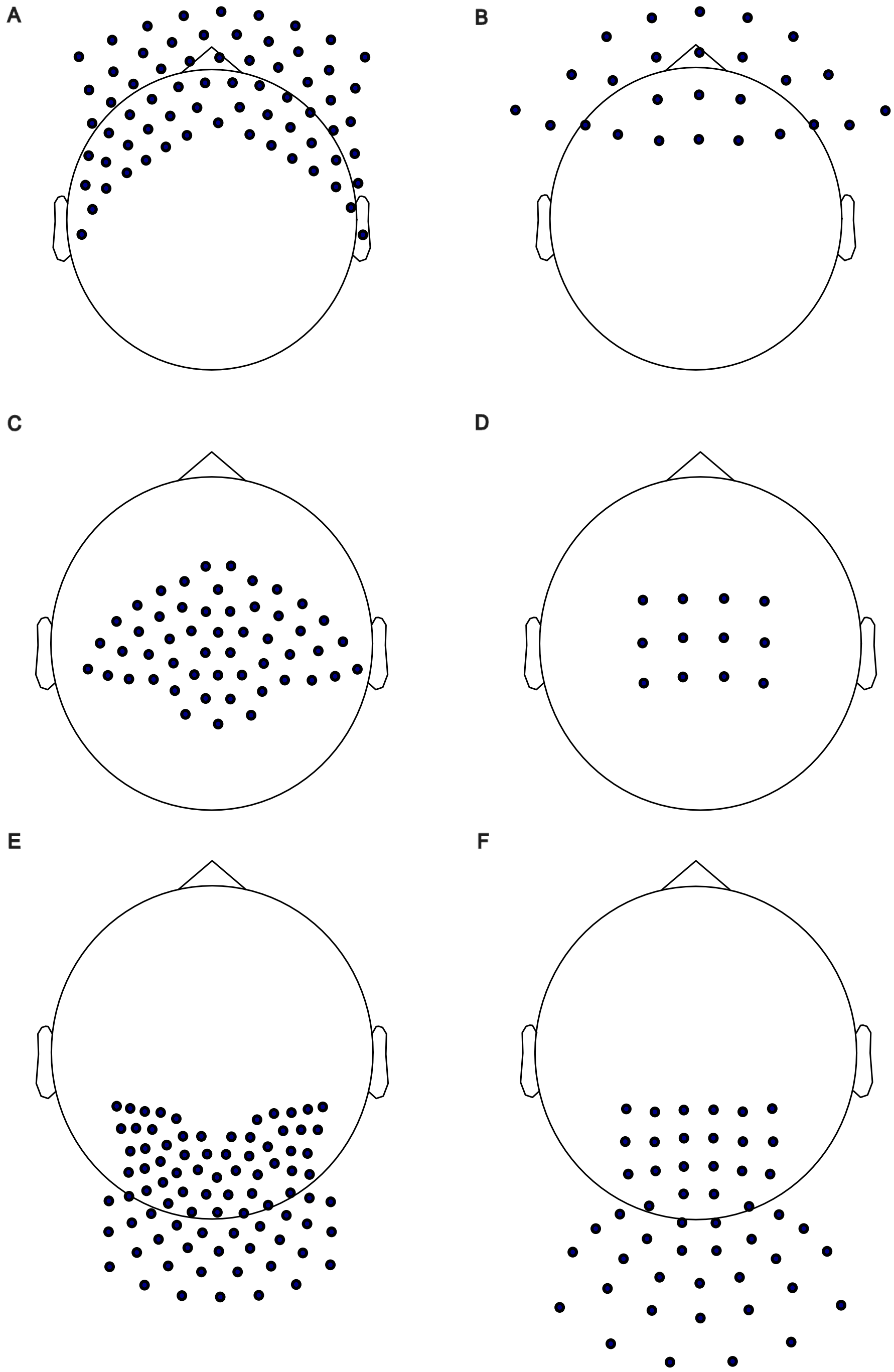


**Supplementary Figure 11.** Partitioning of magnetometer sensors into Regions of Interest (ROIs). Panels A, C, and E display the frontal, central, and posterior regions, respectively, for the OMEGA dataset (recorded with 275 magnetometers). Panels B, D, and F display the corresponding frontal, central, and posterior regions for the NatMEG dataset (recorded with 102 magnetometers).

**Supplementary Table 3.** Summary of meta-analysis results for **unadjusted alpha power peak**

| Study | SMD | SE | CI_Lower | CI_Upper | Weight | *p-value* |
|---|---|---|---|---|---|---|
| San Diego | 0.635 | 0.4309 | -0.21 | 1.479 | 2.89 | |
| Turku | 0 | 0.46547 | -0.912 | 0.912 | 2.48 | |
| Iowa | 0.788 | 0.1857 | 0.424 | 1.152 | 15.58 | |
| New Mexico | 0.689 | 0.30033 | 0.1 | 1.278 | 5.96 | |
| OMEGA | 0.667 | 0.09654 | 0.477 | 0.856 | 57.64 | |
| NatMEG | 0.556 | 0.18647 | 0.19 | 0.921 | 15.45 | |
| Overall (RE Model) | 0.652 | 0.0733 | 0.509 | 0.796 | 100 | 5.59e-19 |

**Supplementary Table 4.** Summary of meta-analysis results for **adjusted alpha power peak**

| Study | SMD | SE | CI_Lower | CI_Upper | Weight | *p-value* |
|---|---|---|---|---|---|---|
| San Diego | -0.011 | 0.42063 | -0.835 | 0.813 | 4.82 | |
| Turku | 0.214 | 0.46642 | -0.701 | 1.128 | 3.97 | |
| Iowa | 0.567 | 0.18311 | 0.208 | 0.926 | 19.89 | |
| New Mexico | 0.796 | 0.30311 | 0.201 | 1.39 | 8.76 | |
| OMEGA | 0.223 | 0.09471 | 0.037 | 0.408 | 42.77 | |
| NatMEG | 0.274 | 0.18383 | -0.087 | 0.634 | 19.78 | |
| Overall (RE Model) | 0.34 | 0.0956 | 0.152 | 0.527 | 100 | 3.77e-04 |

**Supplementary Table 5.** Summary of meta-analysis results for **unadjusted alpha peak frequency**

| Study | SMD | SE | CI_Lower | CI_Upper | Weight | *p-value* |
|---|---|---|---|---|---|---|
| San Diego | -0.615 | 0.43029 | -1.459 | 0.228 | 4.23 | |
| Turku | -1.04 | 0.48733 | -1.996 | -0.085 | 3.33 | |
| Iowa | -0.882 | 0.18703 | -1.248 | -0.515 | 18.92 | |
| New Mexico | -0.16 | 0.29226 | -0.733 | 0.413 | 8.73 | |
| OMEGA | -0.842 | 0.09774 | -1.033 | -0.65 | 45.93 | |
| NatMEG | -0.63 | 0.18745 | -0.998 | -0.263 | 18.85 | |
| Overall (RE Model) | -0.747 | 0.0905 | -0.924 | -0.57 | 100 | 1.47e-16 |

**Supplementary Table 6.** Summary of meta-analysis results for **adjusted alpha peak frequency**

| Study | SMD | SE | CI_Lower | CI_Upper | Weight | *p-value* |
|---|---|---|---|---|---|---|
| San Diego | -0.887 | 0.194 | -1.7503 | -0.0237 | 8.18 | |
| Turku | -0.917 | 0.2329 | -1.8638 | -0.0279 | 7.12 | |
| Iowa | -0.4518 | 0.332 | -0.8087 | -0.0949 | 21.45 | |
| New Mexico | -1516 | 0.854 | -0.4167 | 0.7289 | 14.05 | |
| OMEGA | -0.8105 | 0.0095 | -1.0016 | -0.6194 | 28.16 | |
| NatMEG | -0.607 | 0.035 | -0.9738 | -0.2403 | 21.05 | |
| Overall (RE Model) | -0.5689 | 0.1458 | -0.8547 | -0.2831 | 100 | 9.58e-05 |

**Supplementary Table 7.** Summary of meta-analysis results for **unadjusted beta power peak**

| Study | SMD | SE | CI_Lower | CI_Upper | Weight | *p-value* |
|---|---|---|---|---|---|---|
| San Diego | 0.868 | 0.43968 | 0.007 | 1.73 | 5.01 | |
| Turku | -0.31 | 0.46746 | -1.227 | 0.606 | 4.48 | |
| Iowa | 0.455 | 0.18211 | 0.098 | 0.812 | 20.83 | |
| New Mexico | 0.604 | 0.29836 | 0.019 | 1.188 | 9.92 | |

| | | | | | | |
|---|---|---|---|---|---|---|
| OMEGA | 0.694 | 0.09671 | 0.504 | 0.883 | 39.25 | |
| NatMEG | 0.334 | 0.18425 | -0.027 | 0.695 | 20.51 | |
| Overall (RE Model) | 0.525 | 0.1028 | 0.324 | 0.727 | 100 | 3.26e-07 |

**Supplementary Table 8.** Summary of meta-analysis results for **adjusted beta power peak**

| Study | SMD | SE | CI_Lower | CI_Upper | Weight | *p-value* |
|---|---|---|---|---|---|---|
| San Diego | -0.134 | 0.42109 | -0.96 | 0.691 | 8.74 | |
| Turku | -0.384 | 0.46851 | -1.302 | 0.534 | 7.46 | |
| Iowa | -0.348 | 0.18136 | -0.704 | 0.007 | 21.17 | |
| New Mexico | 0.265 | 0.29307 | -0.31 | 0.839 | 13.91 | |
| OMEGA | 0.356 | 0.09507 | 0.17 | 0.543 | 27.68 | |
| NatMEG | -0.036 | 0.183 | -0.395 | 0.323 | 21.05 | |
| Overall (RE Model) | 0.014 | 0.1466 | -0.274 | 0.301 | 100 | 9.25e-01 |

**Supplementary Table 9.** Summary of meta-analysis results for **unadjusted beta peak frequency**

| Study | SMD | SE | CI_Lower | CI_Upper | Weight | *p-value* |
|---|---|---|---|---|---|---|
| San Diego | 0.142 | 0.42114 | -0.684 | 0.967 | 2.9 | |
| Turku | 0.653 | 0.47421 | -0.276 | 1.583 | 2.29 | |
| Iowa | -0.074 | 0.18034 | -0.427 | 0.28 | 15.82 | |
| New Mexico | 0.134 | 0.29212 | -0.439 | 0.706 | 6.03 | |
| OMEGA | -0.104 | 0.09453 | -0.29 | 0.081 | 57.59 | |
| NatMEG | -0.072 | 0.18304 | -0.431 | 0.286 | 15.36 | |
| Overall (RE Model) | -0.056 | 0.0717 | -0.196 | 0.085 | 100 | 4.37e-01 |

**Supplementary Table 10.** Summary of meta-analysis results for **adjusted beta peak frequency**

| Study | SMD | SE | CI_Lower | CI_Upper | Weight | *p-value* |
|---|---|---|---|---|---|---|
| San Diego | -0.552 | 0.4284 | -1.392 | 0.287 | 2.81 | |
| Turku | 0.4283 | 0.4692 | -0.4914 | 1.348 | 2.34 | |
| Iowa | -0.2143 | 0.1807 | -0.5685 | 0.1398 | 15.79 | |
| New Mexico | -0.0603 | 0.2919 | -0.6324 | 0.5117 | 6.05 | |
| OMEGA | -0.0482 | 0.0945 | -0.2334 | 0.137 | 57.77 | |
| NatMEG | -0.3132 | 0.1841 | -0.6741 | 0.0476 | 15.22 | |
| Overall (RE Model) | -0.1185 | 0.0718 | -0.2593 | 0.022 | 100 | 0.0989 |

**Supplementary Table 11.** Summary of meta-analysis results for **exponent**

| Study | SMD | SE | CI_Lower | CI_Upper | Weight | *p-value* |
|---|---|---|---|---|---|---|
| San Diego | 0.317 | 0.42321 | -0.512 | 1.147 | 8.17 | |
| Turku | 1.144 | 0.49177 | 0.18 | 2.108 | 6.45 | |
| Iowa | 0.307 | 0.18112 | -0.048 | 0.662 | 21.82 | |
| New Mexico | 0.859 | 0.30495 | 0.261 | 1.457 | 12.94 | |
| OMEGA | 0.45 | 0.09543 | 0.263 | 0.637 | 29.9 | |
| NatMEG | 0.972 | 0.19344 | 0.593 | 1.351 | 20.73 | |
| Overall (RE Model) | 0.614 | 0.1383 | 0.343 | 0.885 | 100 | 9.04e-06 |

**Supplementary Table 12.** Summary of meta-analysis results for **offset**

| Study | SMD | SE | CI_Lower | CI_Upper | Weight | *p-value* |
|---|---|---|---|---|---|---|
| San Diego | 1.021 | 0.44677 | 0.146 | 1.897 | 6.18 | |
| Turku | 0.376 | 0.46839 | -0.542 | 1.295 | 5.69 | |
| Iowa | 0.639 | 0.18387 | 0.279 | 1 | 21.68 | |
| New Mexico | 0.811 | 0.30355 | 0.216 | 1.406 | 11.51 | |
| OMEGA | 0.507 | 0.09568 | 0.319 | 0.694 | 34.6 | |
| NatMEG | 1.06 | 0.19535 | 0.677 | 1.443 | 20.34 | |
| Overall (RE Model) | 0.707 | 0.1196 | 0.473 | 0.942 | 100 | 3.37e-09 |

## References

1. Donoghue T. A Systematic Review of Aperiodic Neural Activity in Clinical Investigations. *European Journal of Neuroscience*. 2025;62(7):e70255. doi:10.1111/EJN.70255

2. Ameen MS, Jacobs J, Schabus M, Hoedlmoser K, Donoghue T. The Temporal Dynamics of Aperiodic Neural Activity Track Changes in Sleep Architecture. *bioRxiv*. Published online January 26, 2024:2024.01.25.577204. doi:10.1101/2024.01.25.577204
